# Serum miRNAs are potential biomarkers for the detection of disc degeneration, among which *miR‐26a‐5p* suppresses Smad1 to regulate disc homeostasis

**DOI:** 10.1111/jcmm.14544

**Published:** 2019-07-23

**Authors:** Yunshan Fan, Lan Zhao, Wanqing Xie, Dan Yi, Shisheng He, Di Chen, Jian Huang

**Affiliations:** ^1^ Department of Orthopedics, Shanghai Tenth People's Hospital Tongji University School of Medicine Shanghai China; ^2^ Department of Orthopedic Surgery Rush University Medical Center Chicago IL USA

**Keywords:** circulating miRNA, disc degeneration, *miR‐26a‐5p*, miRNA‐Seq, Smad1, VEGF

## Abstract

Disc degeneration is a common clinical condition in which damaged discs cause chronic pain; however, a laboratory diagnosis method for its detection is not available. As circulating miRNAs have potential as biomarkers, their application in disc degeneration has not been explored. Here, we prepared serum miRNAs from a mouse disc degeneration model and performed miRNA‐Seq and quantitative PCR to characterize disc degeneration–associated miRNAs. We identified three miRNAs, including *miR‐26a‐5p*, *miR‐122‐5p* and *miR‐215‐5p*, undergoing perturbation during the pathogenesis of disc degeneration. Specifically, the levels of *miR‐26a‐5p* in the serum demonstrated steady increases in the model of disc degeneration, compared with those in the pre‐injury samples of younger age or compared with normal controls of the same age but without disc degeneration, whereas the miRNAs *miR‐122‐5p and miR‐215‐5p* exhibited lower expression in post‐injury samples than in their counterparts without the surgery. Moreover, we found that *miR‐26a‐5p* targets Smad1 expression, and Smad1 negatively regulates *Vegfa* expression in disc cells, and thus, *miR‐26a‐5p* promotes disc degeneration. In summary, we established a method that consistently profiles circulating miRNAs and identified multiple miRNAs as promising biomarkers for disc degeneration, among which *miR‐26a‐5p* enhances VEGF expression during disc degeneration through targeting Smad1 signalling.

## 1 INTRODUCTION

Low back pain (LBP) is a leading cause of disability for people in their prime of life, and thus, it is a medical problem imposing a heavy socio‐economic burden.^1^, ^2^ In the United States, LBP is the second most common reason for patients to visit a physician, and the costs related to LBP are estimated to range from $84.1 billion to $624.8 billion.^3^ Being part of the general degeneration process of human beings in ageing, intervertebral disc degeneration (IVDD) is considered as a major cause for LBP,^4^, ^5^ responsible for about 40% LBP in humans.^5^ The intervertebral disc is located between two vertebral bodies and is an avascular cartilaginous structure composed of endplate, annulus fibrosus (AF) and nucleus pulposus (NP). A number of risk factors are associated with the early onset of disc degeneration, such as genetic predisposition, environmental exposure, strenuous activities and smoking.^4^ At present, the diagnosis of disc degeneration mainly depends on imaging studies like MRI.^6^ The changes of water content of intervertebral disc can be detected by MRI. However, it remains difficult to measure the alteration of matrix at earlier stages of disc degeneration.^6^ Furthermore, only patients with severe LBP seek for a MRI examination, rendering earlier detection of disc degeneration by MRI not applicable.

MicroRNAs (miRNAs) are endogenous non‐coding single‐strand RNAs about 23 nucleotides in length. They function by pairing with the messenger RNAs (mRNAs) of their target genes to achieve post‐transcriptional repression.^7^ It is predicted that about 60% of protein‐coding genes have conserved base‐pairing sites targeted by miRNAs, suggesting that miRNAs play an important role in biological processes.^8^ Growing evidence suggests that miRNAs help to maintain the robustness of a biological system by reinforcing transcriptional programmes and regulating aberrant transcripts.^9^, ^10^ Since many miRNAs show a spatiotemporal‐specific manner in regulating both physiological and pathological processes, they present potentials to be used as treatment targets and diagnosis biomarkers.^11^ Particularly, extracellular miRNAs in body fluids, like serum, semen, saliva and urine, are now readily to be detected,^12^ due to the fact that miRNAs can be present in a remarkably stable form in blood and other body fluids. Therefore, investigations of circulating miRNAs as biomarkers for the detection of diseases, including cancers and osteoporosis, have been conducted.^13^

The early detection of intervertebral disc degeneration will help to identify the causes of LBP and guide patients to take early precautions to prevent the acceleration of IVDD. If a sensitive and reliable biomarker in the form of circulating miRNAs dysregulated during disc degeneration could be identified, it would be possible to detect disc degeneration through experimental tests at earlier stages. In this study, we applied the deep sequencing technique to profile the miRNAs that have considerable expression levels in serum, and then we used quantitative reverse transcription PCR (RT‐PCR) to characterize the miRNAs that may undergo dysregulation in serum isolated from an injury‐induced disc degeneration mouse model.^14^ In this study, we have identified three dysregulated circulating miRNAs as potential biomarkers to detect disc degeneration by quantitative RT‐PCR. The result and strategy of this experiment can serve as a reference for clinical study. Importantly, our experimental approach showed feasibility and consistency in preparing and profiling miRNAs, which could be used as a reference to set up a protocol for future clinical investigations. Furthermore, our study revealed *miR‐26a‐5p* as a potent suppressor of Smad1 expression, which targets BMP signalling in disc cells to regulate the pathophysiology of disc tissues.

## 2 MATERIALS AND METHODS

### 2.1 Animal studies

The animal protocol of this study has been approved by the local institutional review board, and all procedures were carried out in accordance with the approved guidelines. To prepare serum for miRNA‐Seq, we collect blood samples from the retroorbital sinus of the mice with plain glass capillary (inside diameter: 1.1‐1.2 mm) into an Eppendorf (EP) tube without any anticoagulation treatment. The collected blood samples were left to coagulate at room temperature for about 30 minutes, and any haemolysis samples were abandoned. Then, we centrifuged the blood samples at 1300 *g* for 10 minutes at room temperature and transferred the supernatant to a new EP tube for another centrifugation to remove any residual cells and debris. Final supernatant was collected and stored at −80°C as serum samples to be used for circulating miRNA preparation.

To generate the injury‐induced disc degeneration model, 12‐week‐old C57BL/6 mice were used. The tail disc Co6/7 was exposed by making a 1‐inch longitudinal incision along the lateral side of the tail. Afterwards, we inserted a 26G needle into the disc for about 1.5 mm. The needle was held in the disc for about 30 seconds before withdrawal. Blood samples for quantitative RT‐PCR were collected at the following three time‐points: before surgery, 2 weeks after puncture and 4 weeks after puncture. After final serum collection (4 weeks after the puncture), the mice were killed and tails were harvested for radiological and histological studies.

### 2.2 µCT and histology

The tail tissues were fixed in 4% paraformaldehyde for 72 hours and then stored in 70% ethanol before µCT measurement or histological processing. A µCT35 desktop cone‐beam scanner (Scanco Medical) was used to scan the mouse tail at a resolution of 12 µm with a 55 kVp source and a 145 µAmp current. We performed three‐dimensional (3D) reconstruction on Co4‐Co7 vertebra. After CT reconstruction, we measured the intervertebral height of punctured disc normalized to adjacent vertebral body lengths using ImageJ. For comparison, the intervertebral heights of intact discs were also measured (n = 5).

After µCT examination, the tail tissues were decalcified with 10% formic acid for 3 weeks and then processed with a tissue processor (Excelsior™ AS Tissue Processor). The tail tissues were dehydrated with graded ethanol, immersed with xylene and embedded in paraffin. Serial sectioning was performed at thickness of 3 µm within the midsagittal region of the intervertebral disc. The sections were stained with Alcian blue & Orange G staining for histological analysis. Immunohistochemistry (IHC) was performed as previously reported,^15^ with sections incubated with 1:50 anti‐VEGF antibody (R&D Systems) or 1:200 anti‐Smad1 antibody (Abcam).

### 2.3 Serum miRNA isolation and miRNA‐Seq

After serum collection, RNA isolation was performed with miRNeasy Serum/Plasma Kit (QIAGEN) according to the manufacturer's protocol with minor modifications. Specifically, we mixed 1 mL Qiazol reagent with 200 µL serum sample with 5.6 × 10^8^ copies of cel‐miR‐39 added as spike‐in control to monitor RNA recovery and reverse transcription efficiency. A volume of 30 µL RNase‐free water was used to elute RNA. RNA sequencing was performed and analysed by Admera Health. Small RNA sequencing libraries were prepared using NEBNext Multiplex Small RNA Library Prep Set according to the manufacturer's instruction. Briefly, small RNA molecules were ligated to 3′ and 5′ adapters and reverse‐transcribed into complementary DNAs (cDNAs). Then, the cDNAs were amplified by PCR to selectively enrich fragments with adapters on both ends. After that, the cDNAs were size‐fractioned and purified on a 6% polyacrylamide gel electrophoresis for the construction of RNA libraries, which were used for deep sequencing. The raw read counts were normalized into counts per million (CPM) and included into cluster analysis.

### 2.4 Quantitative RT‐PCR assays for the detection of circulating miRNAs

Through analysis of the miRNA sequencing results, we selected the miRNA candidates with higher levels (>100 sequences mapping to the mature miRNA across all the samples) in serum. For qRT‐PCR detection of these miRNAs, we used qScript microRNA cDNA Synthesis Kit (Quanta BioSciences) to synthesize cDNAs from isolated miRNAs according to the manufacturer's instructions. Briefly, we performed poly(A) tailing reaction to polyadenylate the small RNAs and then used an oligo‐dT adapter primer provided in the kit to reverse transcribe the RNAs into cDNAs. To quantify the amount of a specific miRNA, we designed a specific forward primer for each miRNA and used a universal reverse primer to perform a SYBR Green qRT‐PCR. We also measured the spike‐in control cel‐miR‐39, which was used as an internal control for the PCR experiments. PCR assays for each miRNA were performed in samples with a number of four to seven. The selected miRNAs and the primers for the detection of these miRNAs via qRT‐PCR were listed in Table 1. Particularly, the universal PCR primer (5′ GCA TAG ACC TGA ATG GCG GTA 3′) is paired with a specific primer for each miRNA in PCRs. For the spike‐in control cel‐miR‐39, the specific primer is 5′ TCA CCG GGT GTA AAT CAG CT 3′.

**Table 1:** List of serum miRNAs selected for quantitative RT‐PCR characterization and their specific primer sequences

miRNAs	Primer sequence
*mmu‐miR‐1a‐3p*	5′ GCC TGG AAT GTA AAG AAG TAT GTA T 3′
*mmu‐let‐7a‐5p*	5′ CCG AGC TGA GGT AGT AGG TTG TATA 3′
*mmu‐let‐7c‐5p*	5′ CCG AGC TGA GGT AGT AGG TTG TAT G 3′
*mmu‐miR‐26a‐5p*	5′ TTC AAG TAA TCC AGG ATA GGC T 3′
*mmu‐miR‐100‐5p*	5′ AAC CCG TAG ATC CGA ACT TG 3′
*mmu‐miR‐122‐5p*	5′ TGG AGT GTG ACA ATG GTG TTT 3′
*mmu‐miR‐148a‐3p*	5′ CCT CAG TGC ACT ACA GAA CTT TG 3′
*mmu‐miR‐215‐5p*	5′ CCA TGA CCT ATG ATT TGA CAG AC 3′
*mmu‐miR‐126a‐3p*	5′ TCG TAC CGT GAG TAA TAA TGC G 3′
*mmu‐miR‐451a*	5′ GGA AAC CGT TAC CAT TAC TGA GT 3′
*mmu‐miR‐128‐3p*	5′ TCA CAG TGA ACC GGT CTC T 3′

### 2.5 Disc cell isolation and culture

We harvested tails from 1‐month‐old C57BL/6 mice and isolated disc cells for in vitro study. Scalpels were used to dissect soft tissues from tails to expose discs. Then, we resected discs and separated NP and AF tissues with microscissors and forceps under a microscope. Separately, NP and AF tissues were cut into small pieces and digested with 0.2% pronase and 0.025% collagenase P overnight in a humidified atmosphere of 5% CO_2_ at 37°C. NP and AF cells were collected by centrifugation at 250 *g* for 10 minutes under room temperature and suspended in culture medium. Dulbecco's modified Eagle's medium (DMEM)/Ham's F‐12 (1:1) supplemented with 20% foetal bovine serum (FBS), and 1% penicillin and streptomycin were chosen as culture medium. The obtained cell suspensions were seeded onto 10 mm culture dishes. We changed culture medium every 72 hours and passaged cells into new dishes when they reached a confluence at about 70%‐80%. Full population of NP and AF cells at passage 2‐4 were used for in vitro experiments such as Western blotting, qRT‐PCR and osteoblast differentiation assays.

### 2.6 Transfection, Western blots, reporter assays and staining for alkaline phosphatase activity

Control siRNA and *Smad1* siRNA were ordered from Sigma. The *miR‐26a‐5p* mimic and inhibitor were ordered from Dharmacon. Transfection was performed as previously described.^16^ Western blot analysis was performed as described previously,^17^ using antibodies against β‐actin (Sigma‐Aldrich), Smad1 (Cell Signaling), Lef1 (Cell Signaling), β‐catenin (Becton Dickinson) and active β‐catenin (EMD Millipore). Luciferase reporter assays were performed as previously described.^16^ Briefly, the sequences containing the putative binding sites 1 (5′‐atcgagccttgcatgTACTTGAA‐3′) and 2 (5′‐aaggagccacgataaTACTTGAc‐3′) in the 3′‐UTR of Smad1 were inserted into the cloning site of the CMV‐Luc2 reporter vector. To mutate the binding sites, the seed sequences (the capitalized parts in above sequences) for the *miR‐26a‐5p‐Smad1* interaction were deleted. Control siRNA or *miR‐26a‐5p* mimic was cotransfected with CMV‐luc2 plasmids as well as Renilla luciferase plasmids into mouse CD45^−^ bone marrow stromal cells. To induce osteoblast differentiation of disc cells, after reaching 100% confluency, cells were cultured in α‐MEM supplemented with 10% FBS, 10 nmol/L dexamethasone, 50 µg/mL ascorbic acid and 10 mmol/L β‐glycerophosphate. Alkaline phosphatase (ALP) staining was performed as previously described.^18^

### 2.7 Statistical analysis

We analysed deep sequencing data by using DESeq package for the statistical environment R.^19^ Unpaired Student's *t* test or ANOVA followed by the Dunnett's multiple comparison test was performed using GraphPad Prism version 6.00 (GraphPad Software) to analyse the difference of miRNA expression levels. *P* < .05 was regarded as statistically significant.

## 3 RESULTS

### 3.1 Establishment of injury‐induced disc degeneration animal model

**Figure 1:**
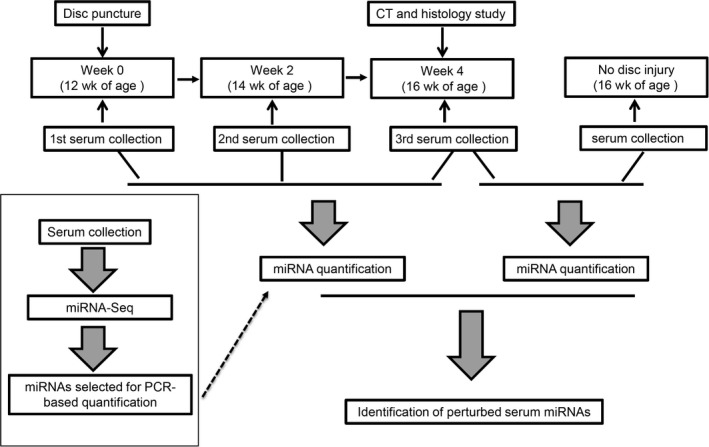
Overview of the study design. Briefly, we extracted serum miRNAs from mice with or without disc injury or at different time‐points post‐injury and performed quantitative RT‐PCR to identify the serum miRNAs with significant perturbations. For the selection of the miRNAs to be tested by PCR, we performed miRNA‐Seq to examine the presence and levels of circulating miRNAs

**Figure 2:**
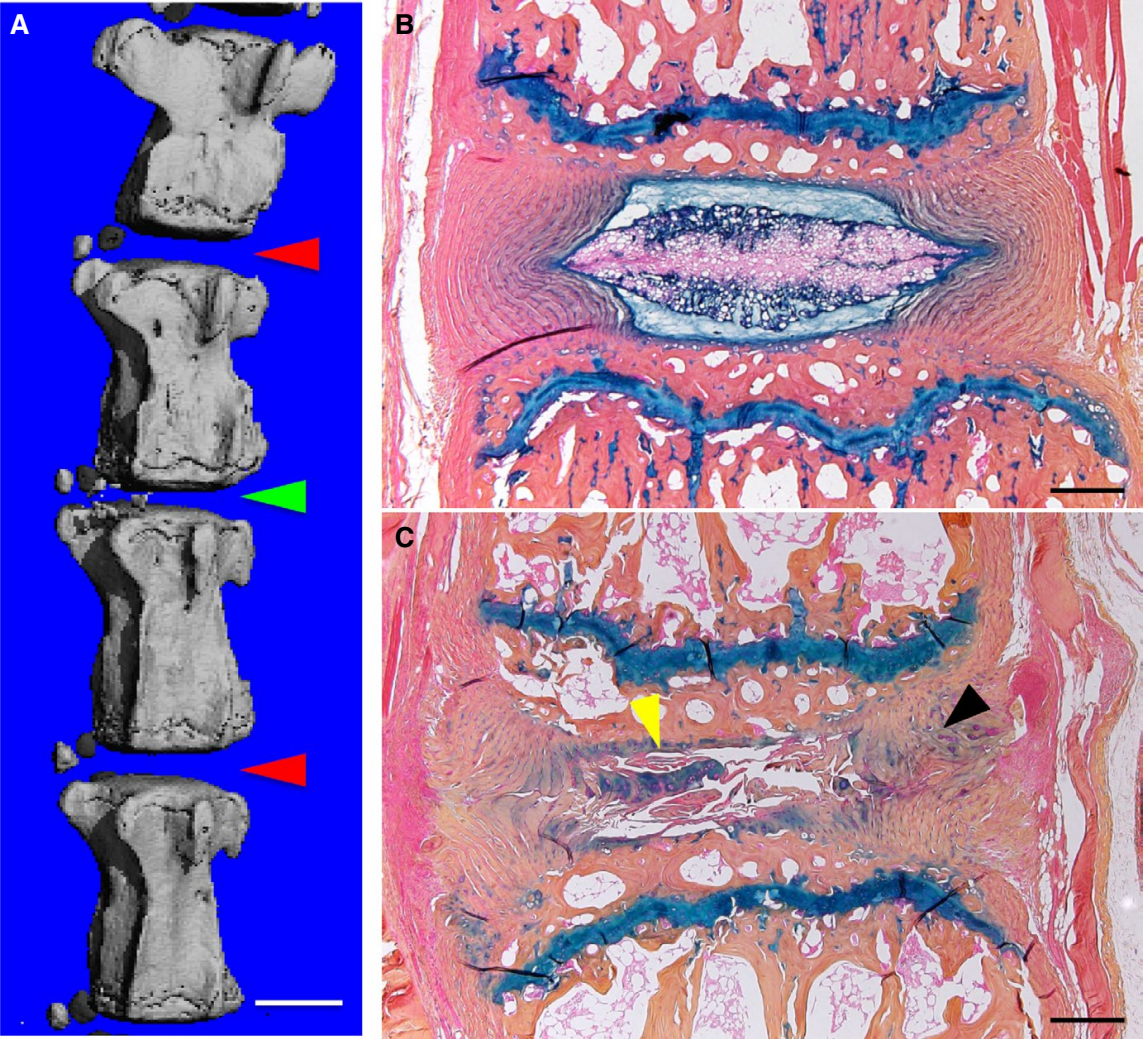
Radiological and histological examinations of injury‐induced disc degeneration in a mouse model. A, µCT 3D reconstruction showed that intervertebral height of the punctured disc was significantly reduced (green arrowhead) compared with that of the control disc (red arrowheads). Scale bar, 1 mm. B, C, Histological comparison of the control disc (B) and the punctured disc (C). Sections were stained with alcian blue/orange G. Black and yellow arrowheads marked abnormal structure of AF and NP in injured disc. Scale bar, 200 μm

To characterize circulating miRNAs in serum during disc degeneration, we generated an injury‐induced disc degeneration animal model and followed a workflow as demonstrated in Figure 1. In brief, we performed disc puncture on 12‐week‐old mice and collected blood samples at the following three time‐points: the day before puncture, 2 and 4 weeks after puncture. After blood collection, the disc tissues were harvested and subjected to radiological and histological examinations. Our micro‐computed tomography (µCT) 3D reconstruction results showed that the intervertebral disc height of the disc with puncture injury was significantly reduced by 27.6% ± 5.1% (*P* = .0158), compared with that of the control disc, indicating a failure of disc structure (Figure 2A). The morphological changes indicative of disc degeneration could be observed on histological sections as well (Figure 2B,C). In the control level without a puncture, the discs had distinct AF and NP regions, the AF was well‐organized, the lamellae were parallel, and the NP displayed a complete structure contained within AF. For the discs that underwent a puncture, the boundary between AF and NP became vague, the AF lost concentric lamellar structure and became serpentine with clefts appearance, and most of the NP was lost and replaced by fibre‐like tissue (Figure 2C). Collectively, our results suggested that this injury‐induced mouse model could successfully recapitulate disc degeneration.

### 3.2 Profiling the levels of circulating miRNAs by miRNA‐Seq

Although circulating miRNAs are remarkably stable in serum and other body fluids, they do have very low levels in serum compared with their existence inside the cells. Thus, the reproducibility and consistence for the detection of circulating miRNAs could be compromised by their extremely low levels, which would cause large variations in the procedures such as miRNA extraction and RT‐PCR. To minimize such technical concerns, we aimed to identify those circulating miRNAs in serum with considerable expression levels and thus we performed miRNA‐Seq to profile the levels of circulating miRNAs. Our miRNA‐Seq yielded an average number of 5 633 523 reads from each sample (16 900 569 reads in total for three samples), among which we detected two major peaks of small RNA reads: a peak at 20‐23 nt consistent with the size of miRNAs and the other peak at 28‐33 nt representing tRNA‐derived fragments (Figure S1A). Further analysis mapped 258 094 reads, about 1.53% of the total reads, to 1177 miRNA sequences, of which each miRNA sequence had at least one read in the sequencing results (Figure S1B). Importantly, the miRNA reads included 299 miRNAs annotated by the miRBase and 843 miRNA isoforms, or isomiRs, with sequence variations that may result from imprecise precursor processing and terminal trimming or tailing.^20^ While most detected miRNAs revealed by the sequencing had known precursors and thus could be classified to known mature miRNAs, we also identified 12 novel miRNAs (Table S1), of which the expression levels were generally lower (<100 sequences mapping to the mature miRNA in any sample). Regarding 843 isomiR reads (Table S2), they constituted a major fraction of the 1177 miRNA reads, suggesting that the majority of serum miRNAs were not identical as those annotated by the miRBase. Thus, it would be interesting to investigate whether these isomiRs were generated inside the cells as a result of alternative processing of pre‐miRNAs, or they underwent any modifications in the extracellular environment. We also calculated the number of the miRNAs that were abundantly expressed in serum and found that about 15% of the miRNAs, including both miRBase‐annotated miRNAs and isomiRs, had relatively higher levels (>100 sequences mapping to the mature miRNA across all the samples), while a vast majority (85%) had a very low profile in serum (Figure S1C). Collectively, our miRNA‐Seq results presented a profile specifying the amounts and the forms of the extracellular miRNAs in serum, which would be helpful for us to determine which miRNAs could be promising candidates for further characterization.

### 3.3 Identification of disc degeneration–associated miRNAs through quantitative RT‐PCR

**Figure 3:**
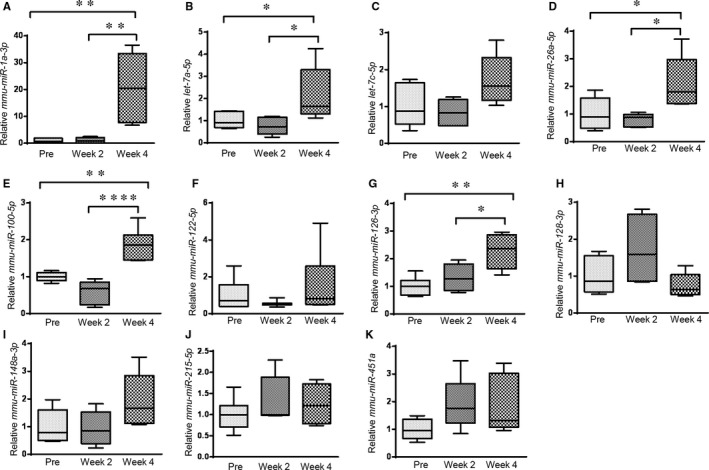
Box plots of 11 selected circulating miRNAs showing their expression levels at three time‐points during the course of disc degeneration. The expression levels of all *miRNAs* were normalized to the spike‐in control (cel‐miR‐39). *P < .05, **P < .01, ****P < .0001, one‐way ANOVA followed by the Dunnett's multiple comparison test

**Figure 4:**
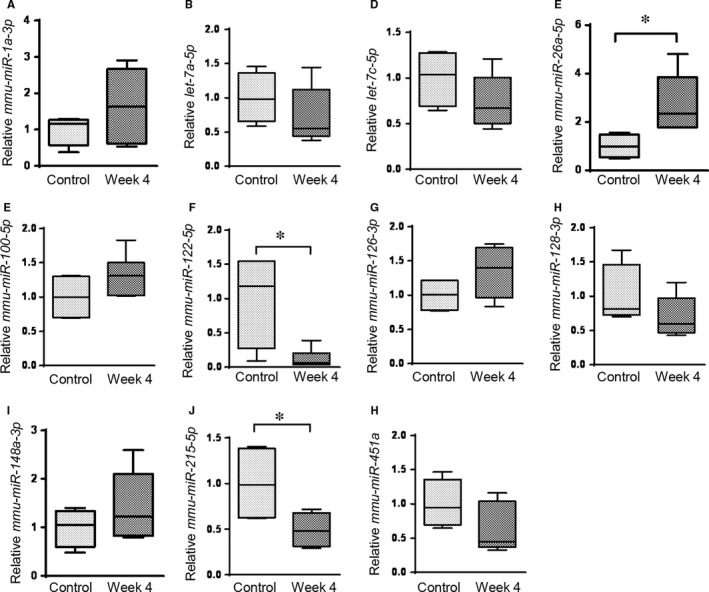
Comparison of 11 selected miRNA expression levels between mice without or with injury at Week 4 post–disc puncture. The expression levels of all miRNAs were normalized to the spike‐in control (cel‐miR‐39). *P < .05, unpaired Student's *t* test

Next, we selected 11 miRNAs that had abundant expression levels (>100 sequences mapping to the mature miRNAs across all the samples) for an analysis of their potentiality as biomarkers for the detection of disc degeneration. We used quantitative RT‐PCR to confirm the expression changes of the 11 circulating miRNAs in serum samples isolated from the mouse disc degeneration model. We compared the expression levels of the circulating miRNAs at different time‐points, for example Week 0, Week 2 and Week 4 post–disc puncture. Interestingly, we found that five of the eleven tested miRNAs, including *miR‐1a‐3p, let‐7a‐5p, miR‐26a‐5p, miR‐100‐5p and miR‐126a‐3p,* increased significantly at Week 4 compared to their levels before the surgery (Week 0), while the other six miRNAs did not change significantly (Figure 3). Since many of the tested miRNAs exhibited an increase in the serum, we suggested that the disc injury was a major event that caused disc degeneration, which might involve remodelling of disc tissues and cell apoptosis to release these miRNAs from the disc tissues into the body fluids. Still, our results could not exclude ageing as a factor to mediate the perturbation of circulating miRNAs, as the mice were getting 4 weeks older when the last collection of blood was completed. To clarify if ageing played a role in regulating circulating miRNA levels and also to further validate the association between the miRNAs and disc degeneration, we extracted serum miRNAs from 16‐week‐old mice without disc puncture surgery, which were the same age to the mice at Week 4 post–disc puncture, and quantified the expression levels of serum miRNAs. Our results demonstrated that most of the miRNAs, including *miR‐1a‐3p*, *let‐7a‐5p*, *let‐7c‐5p, miR‐100‐5p, miR‐126a‐3p, miR‐128‐3p, miR‐148a‐3p* and *miR‐451a*, had no significant changes if the mice underwent surgery or not (Figure 4). Therefore, the increases of these miRNAs, including *miR‐1a‐3p*, *let‐7a‐5p*, *let‐7c‐5p, miR‐126a‐3p* and *miR‐451a*, at Week 4 versus Week 0 were likely due to the ageing but not disc degeneration (Figure S2). We also found that the miRNAs *miR‐122‐5p* and *miR‐215‐5p* decreased in the 16‐week‐old mice with disc injury compared with their counterparts without the surgery, suggesting that these miRNAs may be up‐regulated during ageing (Figure S2) but could be down‐regulated by disc degeneration. Importantly, the serum levels of *miR‐26a‐5p* showed consistent increases in the serum of the 16‐week‐old mice with disc degeneration, compared with those of 16‐week‐old mice without disc degeneration or compared with those in the serum samples isolated from the same mouse at a pre‐injury time‐point (Week 0).

In summary, our results suggested *miR‐26a‐5p* as up‐regulated circulating miRNAs and *miR‐122‐5p* and *miR‐215‐5p* as possibly down‐regulated miRNAs during disc degeneration, among which *miR‐26a‐5p* could hold more potentiality as biomarkers associated with disc degeneration due to its consistent increases in diseased samples.

### 3.4 Characterization of the biological processes that involves the miRNAs

**Figure 5:**
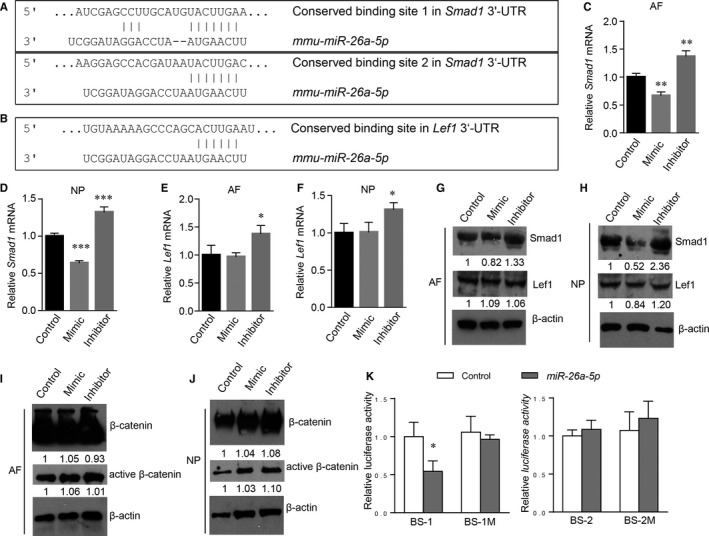
*miR‐26a‐5p* targets Smad1 in both AF and NP cells. A, Two conserved *miR‐26a‐5p*‐binding sites in the 3′‐UTR of *Smad1*. B, A conserved *miR‐26a‐5p*‐binding sites in the 3′‐UTR of *Lef1*. C‐F, Quantitative RT‐PCR analysis of *Smad1* (C, D) and *Lef1 *(E, F) in mouse AF (C, E) or NP (D, F) cells. * *P* < .05, ** *P* < .01, *** *P* < .001, one‐way ANOVA followed by the Dunnett's multiple comparison test. n = 3. Data are shown as the mean ± SD (G‐J) Western blot analysis of Smad1, Lef1, β‐catenin and active β‐catenin proteins in the AF and NP cells transfected with *miR‐26a‐5p* mimic or inhibitor as indicated. The numbers below the blot indicate the relative levels of the denoted protein normalized to β‐actin in each group compared to the control. n = 3 (K) Luciferase activities of 3′‐UTR reporters containing wild‐type or mutated binding site (BS) 1 or 2 with control siRNA or *miR‐26a‐5p* transfection. *P < .05, two‐way ANOVA, n = 3

Next, we investigated the potential roles of the three miRNAs in disc cells by performing an in silico analysis to identify its possible target genes. Of particular, the predicted target genes of *miR‐26a‐5p* attracted our attention, which could be *Smad1* or *Lef1* (Figure 5A,B), two key transcriptional factors in directing osteoblastic differentiation of mesenchymal progenitor cells. As disc tissues are also derived from mesenchymal progenitor cells, we propose that *miR‐26a‐5p* may play an important role in BMP signalling through targeting Smad1 or in Wnt signalling through repressing Lef1, which regulate the pathophysiology of disc tissues. Thus, we isolated AF and NP cells from mouse spine and transfected them with the mimic or the inhibitor of *miR‐26a‐5p*. Examination of mRNA levels in both AF and NP cells revealed that *miR‐26a‐5p* mimic suppressed the expression of Smad1 significantly, and its inhibitor enhanced the expression of Smad1, suggesting that *miR‐26a‐5p* is a bona fide endogenous inhibitor of Smad1 (Figure 5C,D). Similarly, our data of Western blots also demonstrated that *miR‐26a‐5p* suppressed the expression of Smad1 proteins (Figure 5G,H). Although *Lef1* has a binding site in its 3′‐UTR to *miR‐26a‐5p* and its mRNA expression appeared to be boosted by the introduction of *miR‐26a‐5p* inhibitor into the AF and NP cells, we did not found that the transfection of *miR‐26a‐5p* had significant effects on Lef1 expression (Figure 5E,F). We reasoned that the interaction between *Lef1* and *miR‐26a‐5p* could be weaker than that between Smad1 and *miR‐26a‐5p*, because *Smad1* has binding sites with higher affinity to *miR‐26a‐5p*. As our western results demonstrated that the levels of β‐catenin were not significantly altered by the change of *miR‐26a‐5p* expression (Figure 5I,J), we concluded that *miR‐26a‐5p* is unlikely a key player in Wnt/β‐catenin signalling. To further confirm the interaction between *miR‐26a‐5p* and *Smad1* and identify the binding site in the *Smad1* 3′‐UTR responsible for *miR‐26a‐5p‐*mediated repression, we performed luciferase reporter assays and found that transfection of *miR‐26a‐5p* significantly inhibited luciferase expression when the binding site (BS) 1 of *Smad1* to *miR‐26a‐5p* was included in the 3′‐UTR downstream of the luciferase gene, and mutation of BS‐1 abolished this repressive effect (Figure 5K). Thus, BS‐1 could be an important element mediating the suppression of Smad1 by *miR‐26a‐5p*. Inclusion of BS‐2 seemed to have no significant effects, suggesting that it may not be a key binding site for the *miR‐26a‐5p‐Smad1* interaction (Figure 5K). Together, our data suggested that *miR‐26a‐5p* regulates both AF and NP cells through targeting Smad1, a pivotal transcription factor in BMP signalling.^21^

**Figure 6:**
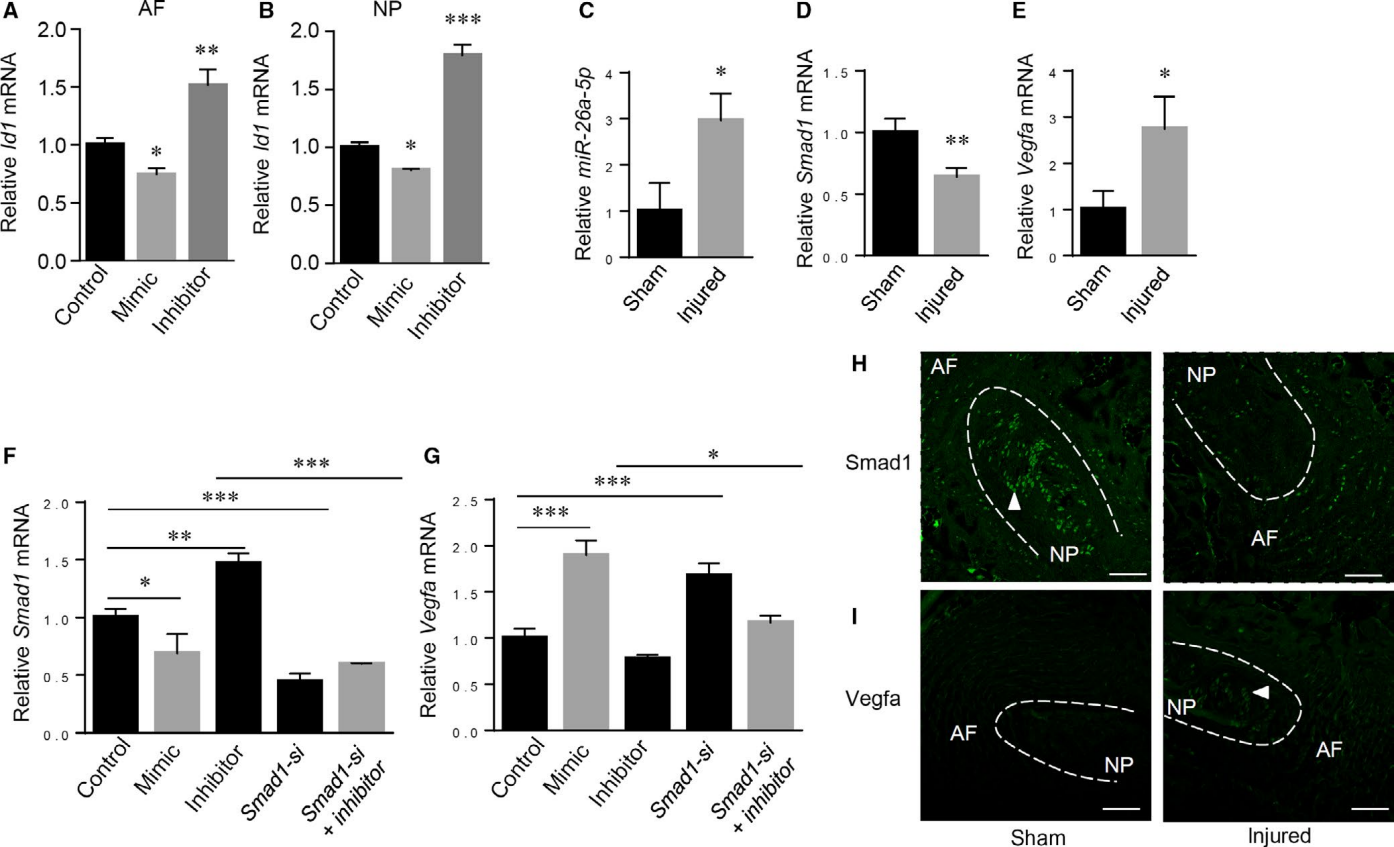
*miR‐26a‐5p* modulates BMP‐Smad1 signalling and Vegfa expression in disc. A, B, Quantitative RT‐PCR analysis of *Id1* in mouse AF or NP cells transfected with *miR‐26a‐5p* mimic or inhibitor as indicated. C‐E, Quantitative RT‐PCR analysis of *miR‐26a‐5p* (C)*, Smad1* (D) and *Vegfa* (E) in sham or injured discs. F, G, Quantitative RT‐PCR analysis of *Smad1* (F) and *Vegfa* (G) in disc cells transfected with *miR‐26a‐5p* mimic or inhibitor, Smad1 siRNAs, or the combination of *Smad1* siRNAs and *miR‐26a‐5p* inhibitor as indicated. H, I, Representative fluorescent immunohistochemistry (IHC) of Smad1 (H) or VEGF‐A (I) expression in sham or injured mouse disc tissues. Arrowhead, IHC‐positive signals. Scale bar, 100 μm. Data are shown as the mean ± SD *P < .05, **P < .01, ***P < .001. Unpaired Student's *t* test for two‐group analysis, or one‐way ANOVA followed by the Dunnett's multiple comparison test for three‐group analysis. n = 3

To further confirm that *miR‐26a‐5p* perturbation affects BMP signalling, we performed qRT‐PCR to analyse the expression of Id1, a well‐documented BMP/Smad1 signalling target gene. We found that Id1 was significantly reduced by *miR‐26a‐5p* mimic and up‐regulated by *miR‐26a‐5p* antagomir (Figure 6A,B), confirming that *miR‐26a*‐*5p* is an endogenous inhibitor of BMP signalling. As disc degeneration involves osteogenesis of disc tissues, we perturbed *miR‐26a‐5p* or Smad1 in disc cells to study their alkaline phosphatase (ALP) activity, a marker of osteogenesis. Although we found that *miR‐26a‐5p*‐mediated Smad1 repression had slightly negative effects on ALP activity in disc cells (Figure S3), ALP activity in the majority of normal disc cells was very low or even negative, suggesting that osteogenesis is not a major event in normal disc cells. This is consistent with a recent study, which reported increased osteogenic differentiation in degenerated disc cells compared with normal disc cells.^22^ Thus, we focused on other regulatory effects of the BMP/Smad1 pathway on disc degeneration. Besides the well‐documented anabolic effects in disc matrix production,^23^, ^24^ it remains obscure whether Smad1 plays a role in the infiltration of blood vessels in the painful degenerated disc. Thus, we compared the expressions of *miR‐26a‐5p*, Smad1 and VEGF‐A, a potent angiogenic growth factor in both healthy and injured discs. Our results demonstrated that *miR‐26a‐5p* induction (Figure 6C), *Smad1* down‐regulation (Figure 6D) and *Vegfa* increase (Figure 6E) are associated with disc degeneration. To investigate whether *Vegfa* expression in disc cells is dependent on *miR‐26a‐5p*‐mediated inhibition of Smad1, we induced the perturbations of *miR‐26a‐5p* and/or Smad1 in disc cells, and found that *miR‐26a‐5p* mimic and Smad1 knockdown both significantly up‐regulated *Vegfa* mRNA levels, while *miR‐26a‐5p* inhibition reduced *Vegfa* expression. Furthermore, Smad1 depletion by siRNAs prevented *miR‐26a‐5p* inhibitors from down‐regulating *Vegfa* (Figure 6F,G). Thus, our results suggested that *Vegfa* induction in disc degeneration requires negative regulation of Smad1 by *miR‐26a‐5p*. We also performed immunohistochemistry to examine the protein levels of Smad1 and VEGF‐A in the disc tissues and found that Smad1 decreased and VEGF‐A increased in degenerated disc tissues (Figure 6H,I). Collectively, our results confirmed a role of *miR‐26a‐5p* in disc degeneration by promoting VEGF‐A expression through repression of Smad1, which may be key event in the invasion of blood vessels in degenerated disc tissues.

## 4 DISCUSSION

As miRNAs can be present in blood in a remarkably stable form,^13^ circulating miRNAs may serve as prognostic biomarkers and treatment‐response predictors in cancer, cardiovascular diseases, diabetes, Alzheimer's disease and many other disorders.^25^ However, using circulating miRNAs as biomarkers for the detection of disc degeneration has not been well explored. In this study, we performed small RNA sequencing and quantitative RT‐PCR to identify circulating miRNAs that were significantly relevant to disc degeneration. In addition, we have established a methodology including the extraction of circulating miRNAs, as well as subsequent reverse transcription and quantitative RT‐PCR, which can provide a consistent, feasible experimental approach for the quantification of circulating miRNAs. As our study explored a new laboratory diagnosis method for the disc degeneration in a disc degeneration animal model, we recognized that the results of our study were generated from animal studies and therefore could not be indiscriminately transferred to clinical applications, whereas our method would provide a practical reference for future clinical studies.

Mounting evidence indicates that miRNAs play an important role in intervertebral disc degeneration. Previous microarray studies demonstrated that miRNAs showed differential expression between degenerated and normal discs.^26^ Moreover, miRNAs showed tissue‐specific expression patterns in degenerated disc tissues, such as AF and NP.^27^ On the other hand, key components of miRNA pathways could be targeted by degeneration‐related factors, resulting in dysregulations of miRNAs in the process of disc degeneration.^28^ Bioinformatic analyses have predicted that the dysregulated miRNAs in degenerated disc tissues might function in vital signalling pathways associated with disc degeneration, such MAPK, TGF‐β and Wnt pathways.^26^ Also, miRNAs were thought to function in disc degeneration by regulating extracellular matrix (ECM) degradation and NP cell proliferation and apoptosis. Several studies have demonstrated that miRNAs participate in disc degeneration by targeting matrix metalloproteinase like matrix metalloproteinase‐13 (MMP13), matrix metalloproteinase‐14 (MMP14) and a disintegrin and metalloproteinase with thrombospondin motifs 5 (ADAMTS5), which contribute to the breakdown of extracellular matrix of discs.^28^, ^29^, ^30^ Besides direct regulation of the matrix‐degrading enzymes, miRNAs also target the upstream genes regulating ECM degradation, including Runt‐related transcription factor 2 (Runx2) and growth differentiation factor 5 (GDF5).^16^, ^31^ In this study, *miR‐26a‐5p* targets Smad1, which serves as a negative regulator of VEGF‐A expression, to promote angiogenesis in the process of disc degeneration. Collectively, these results suggest that miRNAs play important roles in the pathogenic course of intervertebral disc degeneration.

Specifically, circulating miRNAs identified in our study have been demonstrated to be engaged in multiple biological processes associated with tissue degeneration. For example, *let‐7c‐5p* regulates Fas expression and the sensitivity of Fas‐mediated apoptosis.^32^ A member of miR‐126 family, *mmu‐miR‐126a‐3p,* plays an important role in angiogenesis and vascular repair^33^ and might be critical for neovessel formation in tissue degeneration. Here, our results identified *miR‐26a‐5p* as an endogenous regulator of BMP‐Smad1 signalling in disc cells and a promising biomarker for the detection of disc degeneration, suggesting diagnostic and therapeutic potentials of *miR‐26a‐5p* for disc degeneration. Moreover, our study has tested a feasible approach to identify and confirm circulating miRNAs as potential biomarkers for the detection of diseases.

## CONFLICT OF INTEREST

The authors have no conflicts of interest to declare.

## AUTHOR CONTRIBUTIONS

Jian Huang conceived and supervised the study. Yunshan Fan, Lan Zhao, Wanqing Xie, Dan Yi and Jian Huang performed experiments. Jian Huang, Yunshan Fan and Lan Zhao designed experiments, analysed data and wrote the manuscript. Shisheng He and Di Chen revised the manuscript.

## Data Availability

The data that support the findings of this study are available from the corresponding author upon reasonable request.
