# Supplementary material for: Serum miRNAs are potential biomarkers for the detection of disc degeneration, among which miR‐26a‐5p suppresses Smad1 to regulate disc homeostasis
Source: J Cell Mol Med. 2019 Jul 23;23(10):6679–89. doi: 10.1111/jcmm.14544 (PMC6787501; doi:10.1111/jcmm.14544)
Supplement: Supplementary file 1 [file JCMM-23-6679-s001.pdf]

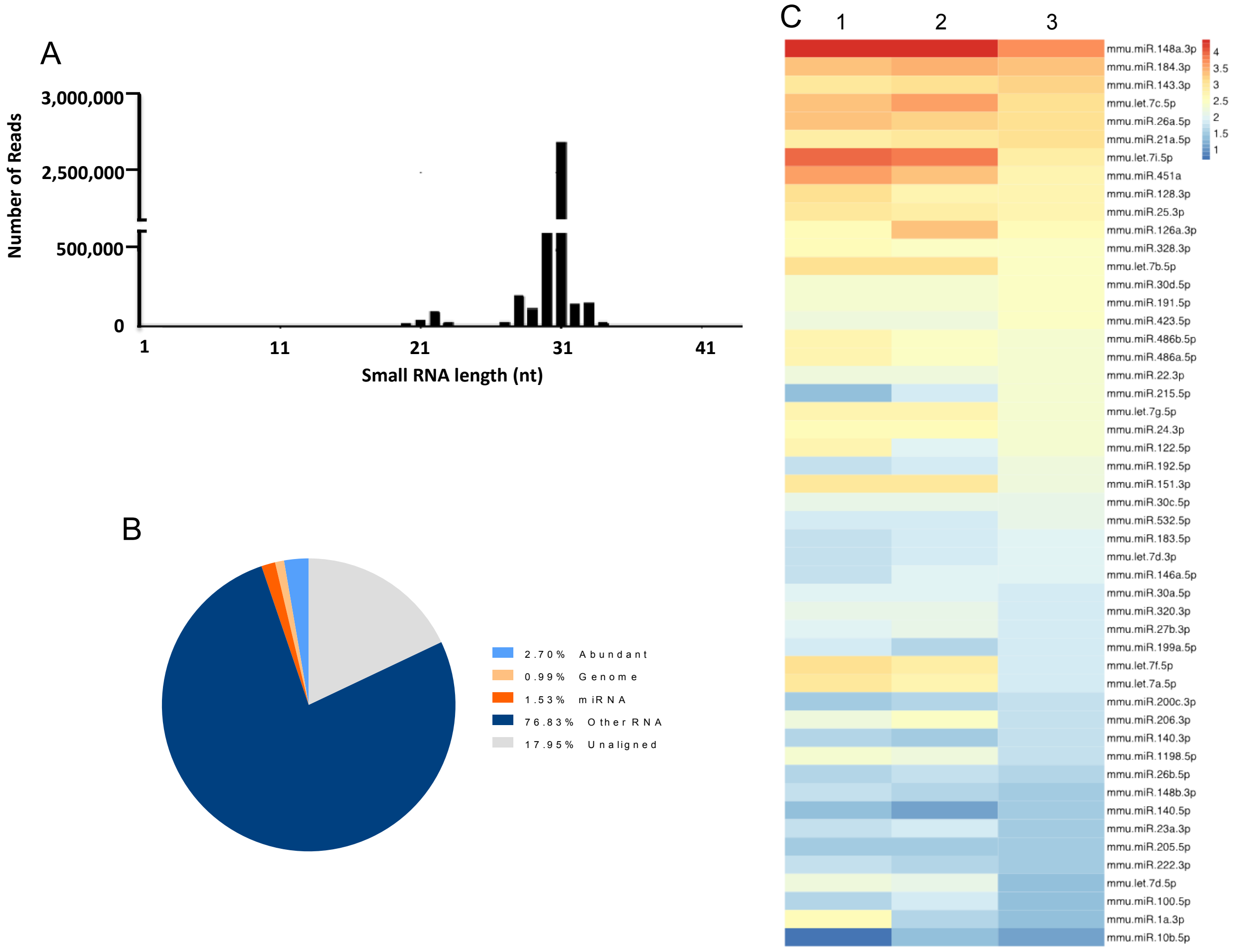

Figure S1. Summary of small RNA sequencing. (A) The peak at 20-23 nt represents miRNAs and the other peak at 28-33 nt represents other RNAs, most of which were derived from tRNAs. (B) Distribution of reads yielded by small RNA sequencing. Reads were aligned against four reference databases (abundant, miRNA, other RNA, and genomic). (C) A heatmap analysis showed the expression levels of circulating miRNAs as revealed by the miR-Seq.

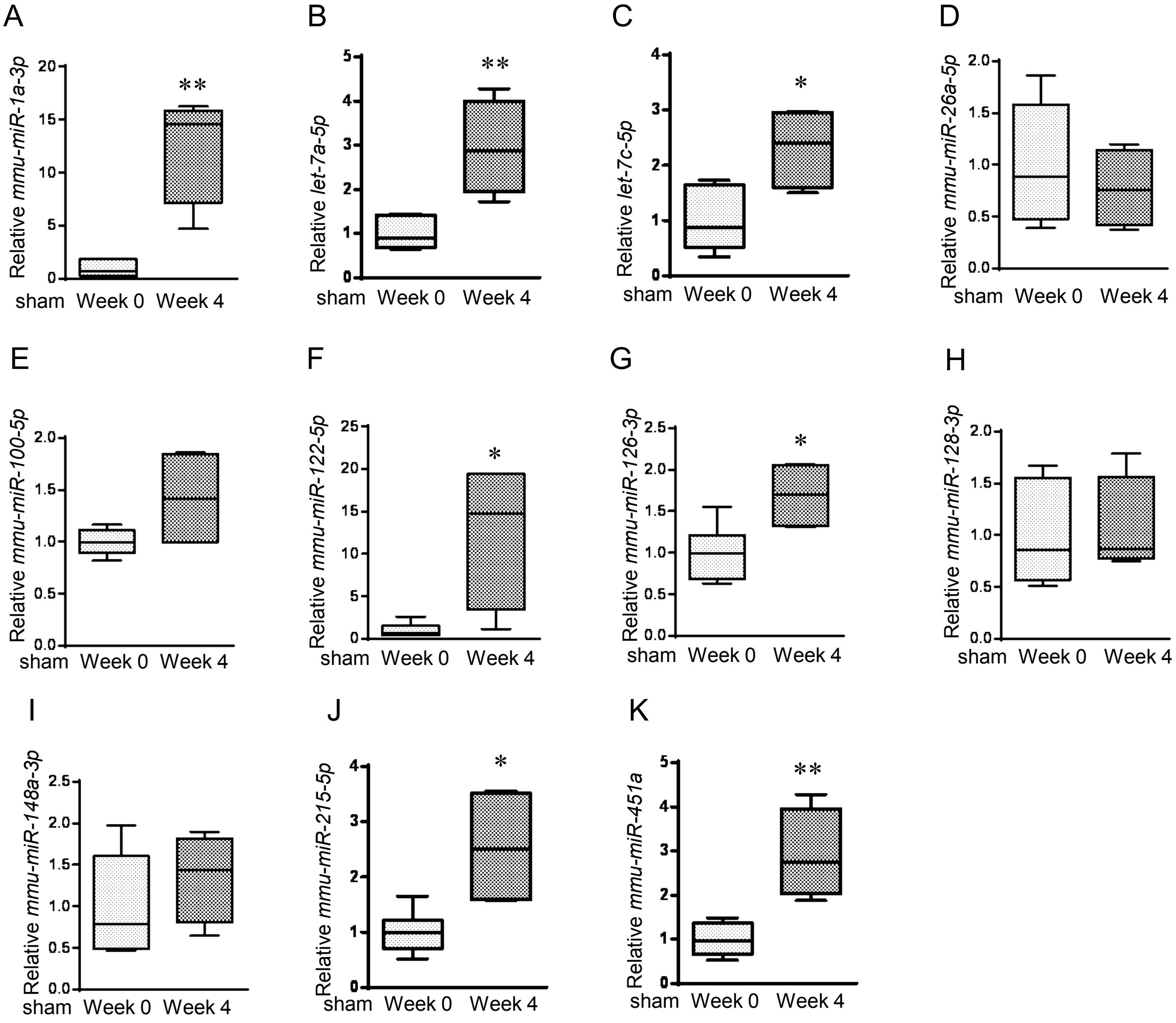

Figure S2 Comparison of 11 selected miRNA expression levels between 12-week-old and 16-week old mice. The expression levels of all miRNAs were normalized to the Spike-In control (cel-miR-39). **P*<0.05, ***P*<0.01, unpaired Student's t-test.

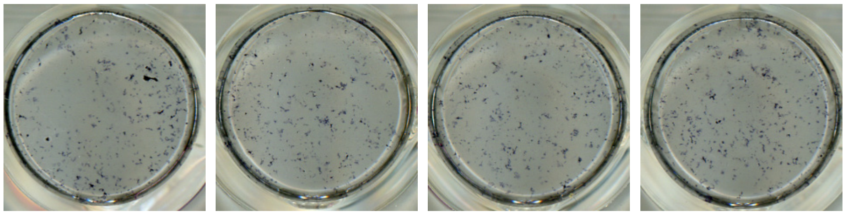

Figure S3 Alkaline phosphatase (ALP) staining of passaged normal disc cells transfected with siRNAs, miRNA mimic or inhibitor as indicated. The numbers indicate relative ALP staining intensities of each group normalized to control.
