# Supplementary material for: Serum miRNAs are potential biomarkers for the detection of disc degeneration, among which miR‐26a‐5p suppresses Smad1 to regulate disc homeostasis
Source: J Cell Mol Med. 2019 Jul 23;23(10):6679–89. doi: 10.1111/jcmm.14544 (PMC6787501; doi:10.1111/jcmm.14544)
Supplement: Supplementary file 2 [file JCMM-23-6679-s002.pdf]

Table S1. List of twelve novel miRNAs identified by miR-Seq.

miR_ID	chr	strand	mature_loci	mature miR
1_10_novelMiR_0	chr1	+	63179111-63179132	ggaggattatgtgtgacagaca
1_15_novelMiR_0	chr6	-	47788159-47788180	ggctggtccgaaggtagtgagt
1_20_novelMiR_0	chrX	-	101997613-101997632	ctagtggttaggattcggca
1_20_novelMiR_1	chrX	-	150625230-150625251	tgaggtagtaggctgtacggct
2_8_novelMiR_0	chr18	-	37854604-37854625	ttgtgtctgtctggctcccagt
2_8_novelMiR_2	chr18	-	56469584-56469605	tgaggtaggagattgggctgtt
2_10_novelMiR_1	chr1	-	43739186-43739205	ttcacagtgggtgagttctt
2_12_novelMiR_0	chr3	-	96464014-96464035	cgtatagtggttagtactctgc
2_15_novelMiR_1	chr6	-	115808093-115808114	gacctcttgggatcgcgtctgg
3_16_novelMiR_0	chr7	+	109520120-109520141	tccgaggctagagtcacgctca
3_17_novelMiR_0	chr8	+	13876097-13876117	tccgaggctagagtcacgctc
3_18_novelMiR_0	chr9	+	88553312-88553329	ttctgggctgtagtgcgc
