# Supplementary material for: Serum miRNAs are potential biomarkers for the detection of disc degeneration, among which miR‐26a‐5p suppresses Smad1 to regulate disc homeostasis
Source: J Cell Mol Med. 2019 Jul 23;23(10):6679–89. doi: 10.1111/jcmm.14544 (PMC6787501; doi:10.1111/jcmm.14544)
Supplement: Supplementary file 3 [file JCMM-23-6679-s003.pdf]

Table S2. Isomirs identified in the miR-Seq. An isomiR ID is in the form of "{start position}_{sequence read}_{precursor ID}"

0_GTAAAGGCTGGGCTTAGACG_mmu-mir-1981
0_GTAAAGGCTGGGCTTAGACGT_mmu-mir-1981
0_GTAAAGGCTGGGCTTAGACGTG_mmu-mir-1981
0_GTAAAGGCTGGGCTTAGACGTGA_mmu-mir-1981
0_GTAAAGGCTGGGCTTAGACGTGG_mmu-mir-1981
0_GTAAAGGCTGGGCTTAGACGTGGCT_mmu-mir-1981
0_GTAAAGGCTGGGCTTAGACGTGGT_mmu-mir-1981
1_CCCATAAAGTAGAAAGC_mmu-mir-142a
1_CCCATAAAGTAGAAAGCACT_mmu-mir-142a
11_AACATTCATTGCTGTCGGTG_mmu-mir-181b-1
11_TGTAAACATCCCCGACTGG_mmu-mir-30d
11_TGTAAACATCCCCGACTGGA_mmu-mir-30d
11_TGTAAACATCCCCGACTGGAA_mmu-mir-30d
11_TGTAAACATCCCCGACTGGAAA_mmu-mir-30d
11_TGTAAACATCCCCGACTGGAAGA_mmu-mir-30d
11_TGTAAACATCCCCGACTGGAAGC_mmu-mir-30d
11_TGTAAACATCCCCGACTGGAAGCC_mmu-mir-30d
11_TGTAAACATCCCCGACTGGAAGCT_mmu-mir-30d
11_TGTAAACATCCCCGACTGGAAGCTTT_mmu-mir-30d
11_TGTAAACATCCCCGACTGGAAGCTTTT_mmu-mir-30d
11_TGTAAACATCCCCGACTGGAAGT_mmu-mir-30d
12_AACCCGTAGATCCGAAATTGT_mmu-mir-100
12_AACCCGTAGATCCGAACT_mmu-mir-100
12_AACCCGTAGATCCGAACTTGC_mmu-mir-100
12_AACCCGTAGATCCGAACTTGT_mmu-mir-100
12_AACCCGTAGATCCGAACTTGTGA_mmu-mir-100
12_AACCCGTAGATCCGAACTTGTGT_mmu-mir-100
12_AACCCGTAGATCCGACCTTGT_mmu-mir-100
12_AACCCGTAGATCCGATCTTGA_mmu-mir-100
12_AACCCGTAGATCCGATCTTGC_mmu-mir-100
12_AACCCGTAGATCCGATCTTGG_mmu-mir-100
12_AACCCGTAGATCCGATCTTGTGA_mmu-mir-100
12_AATGACACGATCACTCCCGT_mmu-mir-425
12_AATGACACGATCACTCCCGTTGAG_mmu-mir-425
12_AATGACACGATCACTCCCGTTGAGC_mmu-mir-425
12_AATGACACGATCACTCCCGTTGAGT_mmu-mir-425
12_ACTCTTTCCCTGTTGCACTAC_mmu-mir-130b
12_TGAGGTAGTAGATTGTATAGC_mmu-let-7a-1
12_TGAGGTAGTAGATTGTATAGTC_mmu-let-7a-1
12_TGAGGTAGTAGGTTGTAT_mmu-let-7a-1
12_TGAGGTAGTAGGTTGTATAG_mmu-let-7a-1
12_TGAGGTAGTAGGTTGTATAGT_mmu-let-7a-1
12_TGAGGTAGTAGGTTGTATAGTTT_mmu-let-7a-1
12_TGAGGTAGTAGGTTGTATGGTC_mmu-let-7a-1
12_TGAGGTAGTAGGTTGTATGGTTA_mmu-let-7a-1
12_TGAGGTAGTAGGTTGTATGGTTAT_mmu-let-7a-1
12_TGAGGTAGTAGGTTGTATGGTTC_mmu-let-7a-1

12_TGCGGGGCTAGGGCTAACAGC_mmu-mir-744
12_TGCGGGGCTAGGGCTAACAGCT_mmu-mir-744
12_TGCGGGGCTAGGGCTAACAGT_mmu-mir-744
13_AACAAGTAATCCAGGATAGGCT_mmu-mir-26a-2
13_AACATTCAACGCTGTCGGT_mmu-mir-181a-1
13_AACATTCAACGCTGTCGGTG_mmu-mir-181a-1
13_AACATTCAACGCTGTCGGTGA_mmu-mir-181a-1
13_AACATTCAACGCTGTCGGTGAGA_mmu-mir-181a-1
13_AAGGAGCTCACAGTCTATTG_mmu-mir-28a
13_AAGGAGCTCACAGTCTATTGA_mmu-mir-28a
13_AAGGGATTCTGATGTTGGTC_mmu-mir-541
13_AAGGGATTCTGATGTTGGTCA_mmu-mir-541
13_CTGACCTATGAATTGACAGC_mmu-mir-192
13_CTGACCTATGAATTGACAGCA_mmu-mir-192
13_CTGACCTATGAATTGACAGCCA_mmu-mir-192
13_CTGACCTATGAATTGACAGCCAAT_mmu-mir-192
13_CTGACCTATGAATTGACAGCCAC_mmu-mir-192
13_CTGACCTATGAATTGACAGCCAT_mmu-mir-192
13_CTGACCTATGAATTGACAGCCATT_mmu-mir-192
13_CTGACCTATGAATTGACAGCCCAT_mmu-mir-192
13_CTGACCTATGAATTGACAGCCT_mmu-mir-192
13_CTGACCTATGAATTGACAGCT_mmu-mir-192
13_CTGACCTATGAATTGACAGT_mmu-mir-192
13_TGAGGTAGTAGGTTGTAT_mmu-let-7c-2
13_TGAGGTAGTAGGTTGTATG_mmu-let-7c-2
13_TGAGGTAGTAGGTTGTATGA_mmu-let-7c-2
13_TGAGGTAGTAGGTTGTATGG_mmu-let-7c-2
13_TGAGGTAGTAGGTTGTATGGT_mmu-let-7c-2
13_TGAGGTAGTAGGTTGTATGGTC_mmu-let-7c-2
13_TGAGGTAGTAGGTTGTATGGTTT_mmu-let-7c-2
13_TGAGGTAGTAGGTTGTGTGA_mmu-let-7c-2
13_TGAGGTAGTAGGTTGTGTGGTTA_mmu-let-7c-2
13_TGAGGTAGTAGGTTGTGTGGTTAT_mmu-let-7c-2
13_TGAGGTAGTAGGTTGTGTGGTTC_mmu-let-7c-2
13_TGAGGTAGTAGGTTGTGTGGTTTT_mmu-let-7c-2
13_TGTAAACATCCTACACTCTC_mmu-mir-30c-2
13_TGTAAACATCCTACACTCTCA_mmu-mir-30c-2
13_TGTAAACATCCTACACTCTCAGA_mmu-mir-30c-2
13_TGTAAACATCCTACACTCTCAGCA_mmu-mir-30c-2
13_TGTAAACATCCTACACTCTCAGCT_mmu-mir-30c-2
13_TGTAAACATCCTACACTCTCAGT_mmu-mir-30c-2
13_TTCAAGTAATCCAGGATAGG_mmu-mir-26a-2
13_TTCAAGTAATCCAGGATAGGCA_mmu-mir-26a-2
13_TTCAAGTAATCCAGGATAGGCC_mmu-mir-26a-2
13_TTCAAGTAATCCAGGATAGGCTAC_mmu-mir-26a-2
13_TTCAAGTAATCCAGGATAGGCTAT_mmu-mir-26a-2
14_AGGACGAGCTAGCTGAGTGCT_mmu-mir-1947
14_TCCCTGAGACCCTAACT_mmu-mir-125b-1
14_TCCCTGAGACCCTAACTT_mmu-mir-125b-1
14_TCCCTGAGACCCTAACTTG_mmu-mir-125b-1

14_TCCCTGAGACCCTAACTTGC_mmu-mir-125b-1
14_TCCCTGAGACCCTAACTTGT_mmu-mir-125b-1
14_TCCCTGAGACCCTAACTTGTG_mmu-mir-125b-1
14_TCCCTGAGACCCTAACTTGTGT_mmu-mir-125b-1
14_TCCTGTACTGAGCTGCCCCG_mmu-mir-486b
14_TCCTGTACTGAGCTGCCCCGA_mmu-mir-486b
14_TCCTGTACTGAGCTGCCCCGAGC_mmu-mir-486b
14_TGACCTATGAATTGACAGCC_mmu-mir-192
14_TGACCTATGAATTGACAGCCA_mmu-mir-192
14_TGACCTATGAATTGACAGCCAC_mmu-mir-192
14_TGACCTATGAATTGACAGCCAG_mmu-mir-192
14_TGACCTATGAATTGACAGCCAGA_mmu-mir-192
14_TGACCTATGAATTGACAGCCAGC_mmu-mir-192
14_TGACCTATGAATTGACAGCCAGT_mmu-mir-192
14_TGACCTATGAATTGACAGCCAT_mmu-mir-192
14_TGACCTATGAATTGACAGCCG_mmu-mir-192
14_TGAGGTAGGAGGTTGTATAG_mmu-let-7e
14_TGAGGTAGGAGGTTGTATAGT_mmu-let-7e
14_TTCAAGTAATTCAGGATAGG_mmu-mir-26b
14_TTCAAGTAATTCAGGATAGGC_mmu-mir-26b
14_TTCAAGTAATTCAGGATAGGCT_mmu-mir-26b
14_TTCAAGTAATTCAGGATAGGTA_mmu-mir-26b
14_TTCAAGTAATTCAGGATAGGTC_mmu-mir-26b
14_TTCAAGTAATTCAGGATAGGTT_mmu-mir-26b
15_AACATTCATTGCTGTCGGTG_mmu-mir-181b-2
15_AACATTCATTGCTGTCGGTGGGA_mmu-mir-181b-2
15_AGAGGTAGTAGATTGTATAGTT_mmu-let-7d
15_AGAGGTAGTAGGTTGCA_mmu-let-7d
15_AGAGGTAGTAGGTTGCAT_mmu-let-7d
15_AGAGGTAGTAGGTTGCATAG_mmu-let-7d
15_AGAGGTAGTAGGTTGCATAGC_mmu-let-7d
15_AGAGGTAGTAGGTTGCATAGT_mmu-let-7d
15_AGAGGTAGTAGGTTGCATAGTTT_mmu-let-7d
15_AGAGGTAGTAGGTTGTATGGT_mmu-let-7d
15_AGAGGTAGTAGGTTGTATGGTT_mmu-let-7d
15_CATCTTACCGGACAGTGCTGGAA_mmu-mir-200a
15_CCCTGAGACCCTAACTTGTGA_mmu-mir-125b-1
15_TAGCAGCACGTAAATATTGGC_mmu-mir-16-1
15_TCCCTGAGGAGCCCTTTGAGCC_mmu-mir-351
15_TGAGGTAGTAGGTTGTAT_mmu-let-7c-1
15_TGAGGTAGTAGGTTGTATG_mmu-let-7c-1
15_TGAGGTAGTAGGTTGTATGA_mmu-let-7c-1
15_TGAGGTAGTAGGTTGTATGG_mmu-let-7c-1
15_TGAGGTAGTAGGTTGTATGGT_mmu-let-7c-1
15_TGAGGTAGTAGGTTGTATGGTC_mmu-let-7c-1
15_TGAGGTAGTAGGTTGTATGGTTT_mmu-let-7c-1
15_TGAGGTAGTAGGTTGTGTGA_mmu-let-7c-1
15_TGAGGTAGTAGGTTGTGTGGC_mmu-let-7c-1
15_TGAGGTAGTAGGTTGTGTGGTTA_mmu-let-7c-1
15_TGAGGTAGTAGGTTGTGTGGTTAA_mmu-let-7c-1

15_TGAGGTAGTAGGTTGTGTGGTTC_mmu-let-7c-1
15_TGTAACAGCAACTCCATGTGA_mmu-mir-194-2
15_TGTAACAGCAACTCCATGTGG_mmu-mir-194-2
15_TTCAAGTAATCCAGGATAGG_mmu-mir-26a-1
15_TTCAAGTAATCCAGGATAGGCA_mmu-mir-26a-1
15_TTCAAGTAATCCAGGATAGGCC_mmu-mir-26a-1
15_TTCAAGTAATCCAGGATAGGCTAT_mmu-mir-26a-1
16_AAACCGTTACCATTACTG_mmu-mir-451a
16_AAACCGTTACCATTACTGAGT_mmu-mir-451a
16_AAACCGTTACCATTACTGAGTTT_mmu-mir-451a
16_AAACCGTTACCATTACTGAGTTTAG_mmu-mir-451a
16_AAACCGTTACCATTACTGAGTTTAGT_mmu-mir-451a
16_TAGCAGCACGTAAATATTGG_mmu-mir-16-2
16_TAGCAGCACGTAAATATTGGC_mmu-mir-16-2
16_TAGCAGCACGTAAATATTGGT_mmu-mir-16-2
16_TATGTGCCTTTGGACTACATCGC_mmu-mir-455
16_TATGTGCCTTTGGACTACATCGT_mmu-mir-455
16_TGAGGTAGTAGATTGTATAGC_mmu-let-7a-2
16_TGAGGTAGTAGATTGTATAGTC_mmu-let-7a-2
16_TGAGGTAGTAGGTTGTAT_mmu-let-7a-2
16_TGAGGTAGTAGGTTGTATA_mmu-let-7a-2
16_TGAGGTAGTAGGTTGTATAG_mmu-let-7a-2
16_TGAGGTAGTAGGTTGTATAGC_mmu-let-7a-2
16_TGAGGTAGTAGGTTGTATAGT_mmu-let-7a-2
16_TGAGGTAGTAGGTTGTATAGTTT_mmu-let-7a-2
16_TGAGGTAGTAGGTTGTATGGTC_mmu-let-7a-2
16_TGAGGTAGTAGGTTGTATGGTTA_mmu-let-7a-2
16_TGAGGTAGTAGGTTGTATGGTTAA_mmu-let-7a-2
16_TGAGGTAGTAGGTTGTATGGTTC_mmu-let-7a-2
16_TGTAAACATCCTACACTCTC_mmu-mir-30c-1
16_TGTAAACATCCTACACTCTCA_mmu-mir-30c-1
16_TGTAAACATCCTACACTCTCAGCT_mmu-mir-30c-1
16_TGTAAACATCCTACACTCTCAGT_mmu-mir-30c-1
16_TGTAAACATCCTTGACTGGAAGCT_mmu-mir-30e
17_TAGCTTATCAGACTGATG_mmu-mir-21a
17_TAGCTTATCAGACTGATGT_mmu-mir-21a
17_TAGCTTATCAGACTGATGTTA_mmu-mir-21a
17_TAGCTTATCAGACTGATGTTG_mmu-mir-21a
17_TAGCTTATCAGACTGATGTTGAC_mmu-mir-21a
17_TAGCTTATCAGACTGATGTTGACA_mmu-mir-21a
17_TAGCTTATCAGACTGATGTTGACT_mmu-mir-21a
18_AGGGGTGCTATCTGTGATTGA_mmu-mir-342
18_AGTTCTTCAGTGGCAAGCTTT_mmu-mir-22
18_TGGAAGACTAGTGATTTTGT_mmu-mir-7a-2
18_TGGAAGACTAGTGATTTTGTT_mmu-mir-7a-2
18_TGGAAGACTAGTGATTTTGTTGTT_mmu-mir-7a-2
18_TTATAAAGCAATGAGACTGA_mmu-mir-340
18_TTATAAAGCAATGAGACTGAT_mmu-mir-340
18_TTATAAAGCAATGAGACTGATC_mmu-mir-340
20_CATGCCTTGAGTGTAGGACCGC_mmu-mir-532

20_CATGCCTTGAGTGTAGGACCGTT_mmu-mir-532
21_ATATAATACAACCTGCTAAGT_mmu-mir-374b
21_TACCCTGTAGAACCGAATTTGA_mmu-mir-10a
21_TACCCTGTAGAACCGAATTTGC_mmu-mir-10a
21_TACCCTGTAGAACCGAATTTGG_mmu-mir-10a
21_TACCCTGTAGATCCGAATT_mmu-mir-10a
21_TACCCTGTAGATCCGAATTT_mmu-mir-10a
21_TACCCTGTAGATCCGAATTTGC_mmu-mir-10a
21_TACCCTGTAGATCCGAATTTGT_mmu-mir-10a
21_TACCCTGTAGATCCGAATTTGTGA_mmu-mir-10a
22_ACCCTGTAGATCCGAATTTGC_mmu-mir-10a
22_ACCCTGTAGATCCGAATTTGG_mmu-mir-10a
22_ACCCTGTAGATCCGAATTTGT_mmu-mir-10a
22_ACCCTGTAGATCCGAATTTGTGA_mmu-mir-10a
22_ACCCTGTAGATCCGAATTTGTGT_mmu-mir-10a
22_GCTCGACTCATGGTTTGAACC_mmu-mir-434
23_ATAGTTGTGTGTGGATGTGTGTA_mmu-mir-669c
23_ATAGTTGTGTGTGGATGTGTGTAT_mmu-mir-669c
23_TGGAAGACTAGTGATTTTGT_mmu-mir-7a-1
23_TGGAAGACTAGTGATTTTGTC_mmu-mir-7a-1
23_TGGAAGACTAGTGATTTTGTT_mmu-mir-7a-1
23_TGGAAGACTAGTGATTTTGTTGTT_mmu-mir-7a-1
23_TGGAAGACTAGTGATTTTGTTT_mmu-mir-7a-1
24_GTGCCTACTGAGCTGAAA_mmu-mir-24-2
24_GTGCCTACTGAGCTGAAAC_mmu-mir-24-2
24_GTGCCTACTGAGCTGAAACAG_mmu-mir-24-2
24_TGAGGTTGGTGTACTGTGTGTG_mmu-mir-672
24_TTGTGCTTGATCTAACCATGC_mmu-mir-218-1
24_TTGTGCTTGATCTAACCATGTGA_mmu-mir-218-2
24_TTGTGCTTGATCTAACCATGTGG_mmu-mir-218-2
24_TTGTGCTTGATCTAACCATGTGT_mmu-mir-218-1
24_TTGTGCTTGATCTAACCATGTGT_mmu-mir-218-2
25_CGTGTATTTGACAAGCTGAGT_mmu-mir-223
25_CGTGTATTTGACAAGCTGAGTT_mmu-mir-223
25_CGTGTATTTGACAAGCTGAGTTGA_mmu-mir-223
25_CGTGTATTTGACAAGCTGAGTTGG_mmu-mir-223
25_CGTGTATTTGACAAGCTGAGTTGGA_mmu-mir-223
25_CGTGTATTTGACAAGCTGAGTTGT_mmu-mir-223
25_CGTGTATTTGACAAGCTGAGTTT_mmu-mir-223
25_TTCCCTTTGTCATCCTATGCCTT_mmu-mir-211
28_TGAGAACTGAATTCCATAGGCA_mmu-mir-146b
28_TGAGAACTGAATTCCATAGGCC_mmu-mir-146b
28_TGAGAACTGAATTCCATAGGCTA_mmu-mir-146b
28_TGAGAACTGAATTCCATAGGCTAT_mmu-mir-146b
28_TGAGAACTGAATTCCATAGGCTG_mmu-mir-146b
28_TGAGAACTGAATTCCATAGGCTGC_mmu-mir-146b
28_TGAGAACTGAATTCCATAGGCTGT_mmu-mir-146b
28_TGAGGGGCAGAGAGCGAGA_mmu-mir-423
28_TGAGGGGCAGAGAGCGAGACC_mmu-mir-423
28_TGAGGGGCAGAGAGCGAGACT_mmu-mir-423

28_TGAGGGGCAGAGAGCGAGACTT_mmu-mir-423
28_TGAGGGGCAGAGAGCGAGACTTC_mmu-mir-423
29_ATGACCTATGATTTGAC_mmu-mir-215
29_ATGACCTATGATTTGACAGA_mmu-mir-215
29_ATGACCTATGATTTGACAGACA_mmu-mir-215
29_ATGACCTATGATTTGACAGACC_mmu-mir-215
29_ATGACCTATGATTTGACAGACT_mmu-mir-215
29_ATGACCTATGATTTGACAGAT_mmu-mir-215
29_TGCCTGTCTACACTTGCTGT_mmu-mir-214
3_AAGGTAGATAGAACAGGTCT_mmu-mir-1839
3_AAGGTAGATAGAACAGGTCTTGT_mmu-mir-1839
3_ACTGGACTTGGAGTCAGA_mmu-mir-378c
3_ACTGGACTTGGAGTCAGAAG_mmu-mir-378c
3_ACTGGACTTGGAGTCAGAAGGCA_mmu-mir-378c
3_ACTGGACTTGGAGTCAGAAGGCT_mmu-mir-378c
3_ACTGGACTTGGAGTCAGAAGGCTTT_mmu-mir-378c
3_CATAAAGTAGAAAGCACT_mmu-mir-142a
3_CATAAAGTAGAAAGCACTA_mmu-mir-142a
3_CATGGAGGTCTCTGTCTGACT_mmu-mir-1843b
3_CATGGAGGTCTCTGTCTGACTT_mmu-mir-1843b
3_TAGCAGCACATCATGGTT_mmu-mir-15b
3_TATGGAGGTCTCTGTCTGACTT_mmu-mir-1843a
3_TCTGGCTCCGTGTCTTCACTC_mmu-mir-149
3_TCTGGCTCCGTGTCTTCACTCC_mmu-mir-149
3_TCTGGCTCCGTGTCTTCACTCT_mmu-mir-149
30_CCCAGTGTTCAGACTACCTGTT_mmu-mir-199a-2
32_TCCTGTACTGAGCTGCCCCG_mmu-mir-486a
32_TCCTGTACTGAGCTGCCCCGA_mmu-mir-486a
32_TCCTGTACTGAGCTGCCCCGAGG_mmu-mir-486a
32_TGAGGTATTAGTTTGTGCTGTT_mmu-let-7j
32_TGGGGTAGTAGTTTGTGCTGTT_mmu-let-7j
34_CTGTACTGAGCTGCCCCGAGGT_mmu-mir-486a
39_TGAGATGAAGCACTGT_mmu-mir-143
39_TGAGATGAAGCACTGTAGCC_mmu-mir-143
39_TGAGATGAAGCACTGTAGCT_mmu-mir-143
39_TGAGATGAAGCACTGTAGCTCT_mmu-mir-143
39_TGAGATGAAGCACTGTAGCTCTT_mmu-mir-143
39_TGAGATGAAGCACTGTAGCTT_mmu-mir-143
39_TGAGATGAAGCACTGTAGCTTT_mmu-mir-143
39_TTTGTTCGTTCGGCTCGCG_mmu-mir-375
39_TTTGTTCGTTCGGCTCGCGT_mmu-mir-375
39_TTTGTTCGTTCGGCTCGCGTG_mmu-mir-375
39_TTTGTTCGTTCGGCTCGCGTGT_mmu-mir-375
4_AACCCGTAGATCCGATCC_mmu-mir-99a
4_AACCCGTAGATCCGATCT_mmu-mir-99a
4_AACCCGTAGATCCGATCTTGC_mmu-mir-99a
4_AACCCGTAGATCCGATCTTGT_mmu-mir-99a
4_AACCCGTAGATCCGATCTTGTAT_mmu-mir-99a
4_AACCCGTAGATCCGATCTTGTGA_mmu-mir-99a
4_AACCCGTAGATCCGATCTTGTGT_mmu-mir-99a

4_ATGGAGGTCTCTGTCTGACT_mmu-mir-1843a
4_CTGGACTTGGAGTCAGAAGGCTTT_mmu-mir-378c
4_TACCCTGTAGAACCGAATT_mmu-mir-10b
4_TACCCTGTAGAACCGAATTTGC_mmu-mir-10b
4_TACCCTGTAGAACCGAATTTGT_mmu-mir-10b
4_TACCCTGTAGAACCGAATTTGTGA_mmu-mir-10b
4_TACCCTGTAGATCCGAATTTGC_mmu-mir-10b
4_TCAGGCTCAGTCCCCTCCCG_mmu-mir-484
4_TCAGGCTCAGTCCCCTCCCGA_mmu-mir-484
4_TCAGGCTCAGTCCCCTCCCGATA_mmu-mir-484
4_TCAGGCTCAGTCCCCTCCCGATT_mmu-mir-484
4_TCAGGCTCAGTCCCCTCCCGTT_mmu-mir-484
40_GTAGTGTTTCCTACTTTATGG_mmu-mir-142a
40_GTAGTGTTTCCTACTTTATGGA_mmu-mir-142a
40_TGGATATGATGACTGATTACCTGAGAAATAATTGATGAAATCTCAAGAAA_mmu-mir-3535
41_CTACAGTATAGATGATGTACT_mmu-mir-144
41_TAACAGTCTACAGCCATGGTCGT_mmu-mir-132
41_TATGTGTTCCTGGCTGGCTTGA_mmu-mir-1198
41_TATGTGTTCCTGGCTGGCTTGGTT_mmu-mir-1198
41_TATGTGTTCCTGGCTGGCTTGT_mmu-mir-1198
42_ACTGGACTTGGAGTCAGAAG_mmu-mir-378a
42_ACTGGACTTGGAGTCAGAAGA_mmu-mir-378a
42_ACTGGACTTGGAGTCAGAAGGA_mmu-mir-378a
42_ACTGGACTTGGAGTCAGAAGGC_mmu-mir-378a
42_ACTGGACTTGGAGTCAGAAGGCA_mmu-mir-378a
42_ACTGGACTTGGAGTCAGAAGGCAT_mmu-mir-378a
42_ACTGGACTTGGAGTCAGAAGGCC_mmu-mir-378a
42_ACTGGACTTGGAGTCAGAAGGCG_mmu-mir-378a
42_ACTGGACTTGGAGTCAGAAGGCGT_mmu-mir-378a
42_ACTGGACTTGGAGTCAGAAGGCT_mmu-mir-378a
42_ACTGGACTTGGAGTCAGAAGGT_mmu-mir-378a
42_CTAGACTGAGGCTCCTTGA_mmu-mir-151
42_CTAGACTGAGGCTCCTTGAG_mmu-mir-151
42_CTAGACTGAGGCTCCTTGAGA_mmu-mir-151
42_CTAGACTGAGGCTCCTTGAGAAA_mmu-mir-151
42_CTAGACTGAGGCTCCTTGAGGA_mmu-mir-151
42_CTAGACTGAGGCTCCTTGAGGAA_mmu-mir-151
42_CTAGACTGAGGCTCCTTGAGGACA_mmu-mir-151
42_CTAGACTGAGGCTCCTTGAGGACC_mmu-mir-151
42_CTAGACTGAGGCTCCTTGAGGACT_mmu-mir-151
42_CTAGACTGAGGCTCCTTGAGGAT_mmu-mir-151
42_CTAGACTGAGGCTCCTTGAGGATT_mmu-mir-151
42_CTAGACTGAGGCTCCTTGAGGT_mmu-mir-151
42_CTAGACTGAGGCTCCTTGAGGTA_mmu-mir-151
42_CTAGACTGAGGCTCCTTGAGGTT_mmu-mir-151
42_CTAGACTGAGGCTCCTTGAGT_mmu-mir-151
42_CTAGACTGAGGCTCCTTGAGTAA_mmu-mir-151
42_CTAGACTGAGGCTCCTTGAGTT_mmu-mir-151
42_TACAGTATAGATGATG_mmu-mir-144
42_TACAGTATAGATGATGTAC_mmu-mir-144

42_TCCCCCAGGTGTGATTCTGATTTGC_mmu-mir-361
42_TCGGATCCGTCTGAGCTTGG_mmu-mir-127
42_TTTGGTCCCCTTCAACCAGCTGT_mmu-mir-133a-1
43_CCCCCAGGTGTGATTCTGATTTGC_mmu-mir-361
43_CCCCCAGGTGTGATTCTGATTTGT_mmu-mir-361
43_CTGGACTTGGAGTCAGAAGGC_mmu-mir-378a
43_CTGGACTTGGAGTCAGAAGGCA_mmu-mir-378a
43_CTGGACTTGGAGTCAGAAGGCC_mmu-mir-378a
43_CTGGACTTGGAGTCAGAAGGCT_mmu-mir-378a
43_TAGACTGAGGCTCCTTGAGGACT_mmu-mir-151
43_TCACAGTGAACCGGTCTCT_mmu-mir-128-1
43_TCACAGTGAACCGGTCTCTC_mmu-mir-128-1
43_TCACAGTGAACCGGTCTCTT_mmu-mir-128-1
43_TCACAGTGAACCGGTCTCTTC_mmu-mir-128-1
43_TCACAGTGAACCGGTCTCTTTAA_mmu-mir-128-1
43_TCACAGTGAACCGGTCTCTTTAT_mmu-mir-128-1
43_TCACAGTGAACCGGTCTCTTTT_mmu-mir-128-1
43_TCACAGTGAACCGGTCTCTTTTAT_mmu-mir-128-1
43_TCACAGTGAACCGGTCTCTTTTT_mmu-mir-128-1
43_TCACAGTGAACCGGTCTCTTTTTT_mmu-mir-128-1
43_TGGCTCAGTTCAGCAGGAAC_mmu-mir-24-1
43_TGGCTCAGTTCAGCAGGAACA_mmu-mir-24-1
43_TTGGTCCCCTTCAACCAGCTGT_mmu-mir-133a-1
44_ATCAACAGACATTAATTGGGCGT_mmu-mir-421
44_CAAGCTCGTGTCTGTGGGTCCGA_mmu-mir-99b
44_TAATACTGCCGGGTAATGATA_mmu-mir-200c
44_TAATACTGCCGGGTAATGATG_mmu-mir-200c
44_TAATACTGCCGGGTAATGATGG_mmu-mir-200c
44_TAATACTGCCGGGTAATGATGGAA_mmu-mir-200c
44_TAATACTGCCGGGTAATGATGGG_mmu-mir-200c
44_TAATACTGCCTGGTAATGATA_mmu-mir-200b
44_TAATACTGCCTGGTAATGATG_mmu-mir-200b
44_TAATACTGCCTGGTAATGATGAC_mmu-mir-200b
44_TAATACTGCCTGGTAATGATGACA_mmu-mir-200b
44_TAATACTGCCTGGTAATGATGACT_mmu-mir-200b
44_TAATACTGCCTGGTAATGATGAT_mmu-mir-200b
44_TACAGTAGTCTGCACATTGGTT_mmu-mir-199a-1
44_TACCACAGGGTAGAACCACG_mmu-mir-140
44_TACCACAGGGTAGAACCACGA_mmu-mir-140
44_TACCACAGGGTAGAACCACGGA_mmu-mir-140
44_TACCACAGGGTAGAACCACGGAA_mmu-mir-140
44_TACCACAGGGTAGAACCACGGAC_mmu-mir-140
44_TACCACAGGGTAGAACCACGGACA_mmu-mir-140
44_TACCACAGGGTAGAACCACGGACC_mmu-mir-140
44_TACCACAGGGTAGAACCACGGACG_mmu-mir-140
44_TACCACAGGGTAGAACCACGGACGT_mmu-mir-140
44_TACCACAGGGTAGAACCACGGACT_mmu-mir-140
44_TACCACAGGGTAGAACCACGGAGT_mmu-mir-140
44_TACCACAGGGTAGAACCACGGATT_mmu-mir-140
44_TGGACGGAGAACTGATA_mmu-mir-184

44_TGGACGGAGAACTGATAAGGGC_mmu-mir-184
44_TGGACGGAGAACTGATAAGGGTT_mmu-mir-184
44_TGGACGGAGAACTGATAAGGGTTT_mmu-mir-184
45_AATACTGCCTGGTAATGATGACA_mmu-mir-200b
45_AATACTGCCTGGTAATGATGACT_mmu-mir-200b
45_ACAAGTCAGGTTCTTGGGACA_mmu-mir-125b-2
45_ACAGTAGTCTGCACATTGGTC_mmu-mir-199a-1
45_ACAGTAGTCTGCACATTGGTT_mmu-mir-199a-1
45_ACAGTAGTCTGCACATTGGTTT_mmu-mir-199a-1
45_ACCACAGGGTAGAACC_mmu-mir-140
45_ACCACAGGGTAGAACCACGG_mmu-mir-140
45_ACCACAGGGTAGAACCACGGA_mmu-mir-140
45_ACCACAGGGTAGAACCACGGAA_mmu-mir-140
45_ACCACAGGGTAGAACCACGGAAA_mmu-mir-140
45_ACCACAGGGTAGAACCACGGAC_mmu-mir-140
45_ACCACAGGGTAGAACCACGGACA_mmu-mir-140
45_ACCACAGGGTAGAACCACGGACC_mmu-mir-140
45_ACCACAGGGTAGAACCACGGACG_mmu-mir-140
45_ACCACAGGGTAGAACCACGGACGT_mmu-mir-140
45_ACCACAGGGTAGAACCACGGACT_mmu-mir-140
45_ATCACATTGCCAGGGAT_mmu-mir-23a
45_ATCACATTGCCAGGGAT_mmu-mir-23b
45_ATCACATTGCCAGGGATT_mmu-mir-23a
45_ATCACATTGCCAGGGATT_mmu-mir-23b
45_ATCACATTGCCAGGGATTACCACT_mmu-mir-23b
45_ATCACATTGCCAGGGATTT_mmu-mir-23a
45_ATCACATTGCCAGGGATTTA_mmu-mir-23a
45_ATCACATTGCCAGGGATTTC_mmu-mir-23a
45_ATCACATTGCCAGGGATTTCCA_mmu-mir-23a
45_GATTCCTGGAAATACTGTTC_mmu-mir-145a
45_GATTCCTGGAAATACTGTTCT_mmu-mir-145a
45_GATTCCTGGAAATACTGTTCTT_mmu-mir-145a
45_TCGTACCGTGAGTAATAATA_mmu-mir-126a
45_TCGTACCGTGAGTAATAATG_mmu-mir-126a
45_TCGTACCGTGAGTAATAATGC_mmu-mir-126a
45_TCGTACCGTGAGTAATAATGCA_mmu-mir-126a
45_TCGTACCGTGAGTAATAATGT_mmu-mir-126a
45_TGGAATGTAAGGAAGTGTGT_mmu-mir-206
45_TGGAATGTAAGGAAGTGTGTG_mmu-mir-206
45_TGGAATGTAAGGAAGTGTGTGA_mmu-mir-206
45_TGGAATGTAAGGAAGTGTGTGT_mmu-mir-206
46_ATTCCTGGAAATACTGTTC_mmu-mir-145a
46_ATTCCTGGAAATACTGTTCT_mmu-mir-145a
46_ATTCCTGGAAATACTGTTCTT_mmu-mir-145a
46_CAGTAGTCTGCACATTGGTT_mmu-mir-199a-1
46_CCACAGGGTAGAACCACGGACT_mmu-mir-140
46_CGTACCGTGAGTAATAATGC_mmu-mir-126a
46_CGTACCGTGAGTAATAATGCG_mmu-mir-126a
46_CGTACCGTGAGTAATAATGCGA_mmu-mir-126a
46_CGTACCGTGAGTAATAATGCGT_mmu-mir-126a

46_CGTACCGTGAGTAATAATGT_mmu-mir-126a
46_CTTTCAGTCGGATGTTTGC_mmu-mir-30a
46_CTTTCAGTCGGATGTTTGCAG_mmu-mir-30a
46_CTTTCAGTCGGATGTTTGCAGTT_mmu-mir-30a
46_TCACATTGCCAGGGATTACCACT_mmu-mir-23b
46_TCAGTGCATGACAGAACT_mmu-mir-152
46_TCAGTGCATGACAGAACTTG_mmu-mir-152
46_TCAGTGCATGACAGAACTTGA_mmu-mir-152
46_TCAGTGCATGACAGAACTTGGA_mmu-mir-152
46_TCAGTGCATGACAGAACTTGGG_mmu-mir-152
46_TCAGTGCATGACAGAACTTGGT_mmu-mir-152
46_TGGAATGTAAAGAAGTATGT_mmu-mir-1a-2
47_AAAAGCTGGGTTGAGAGGG_mmu-mir-320
47_AAAAGCTGGGTTGAGAGGGC_mmu-mir-320
47_AAAAGCTGGGTTGAGAGGGCA_mmu-mir-320
47_AAAAGCTGGGTTGAGAGGGCG_mmu-mir-320
47_AAAAGCTGGGTTGAGAGGGCGAA_mmu-mir-320
47_AAAAGCTGGGTTGAGAGGGCGAAA_mmu-mir-320
47_AAAAGCTGGGTTGAGAGGGCGAAC_mmu-mir-320
47_AAAAGCTGGGTTGAGAGGGCGAAG_mmu-mir-320
47_AAAAGCTGGGTTGAGAGGGCGAAT_mmu-mir-320
47_AAAAGCTGGGTTGAGAGGGCGAGA_mmu-mir-320
47_AAAAGCTGGGTTGAGAGGGCGAGT_mmu-mir-320
47_AAAAGCTGGGTTGAGAGGGCGAT_mmu-mir-320
47_AAAAGCTGGGTTGAGAGGGCGATT_mmu-mir-320
47_AAAAGCTGGGTTGAGAGGGCGT_mmu-mir-320
47_AAAAGCTGGGTTGAGAGGGCGTT_mmu-mir-320
47_AATGTTGCTCGGTGAACCCCT_mmu-mir-409
47_GTGAAATGTTTAGGAC_mmu-mir-203
47_GTGAAATGTTTAGGACCACT_mmu-mir-203
47_GTGAAATGTTTAGGACCACTAA_mmu-mir-203
47_TAACACTGTCTGGTAAAGATG_mmu-mir-141
47_TAACACTGTCTGGTAACGATA_mmu-mir-141
47_TCACAGTGAACCGGTCTCTC_mmu-mir-128-2
47_TCACAGTGAACCGGTCTCTT_mmu-mir-128-2
47_TCACAGTGAACCGGTCTCTTAA_mmu-mir-128-2
47_TCACAGTGAACCGGTCTCTTC_mmu-mir-128-2
47_TCACAGTGAACCGGTCTCTTTA_mmu-mir-128-2
47_TCACAGTGAACCGGTCTCTTTAA_mmu-mir-128-2
47_TCACAGTGAACCGGTCTCTTTAT_mmu-mir-128-2
47_TCACAGTGAACCGGTCTCTTTC_mmu-mir-128-2
47_TCACAGTGAACCGGTCTCTTTCT_mmu-mir-128-2
47_TCACAGTGAACCGGTCTCTTTTA_mmu-mir-128-2
47_TCACAGTGAACCGGTCTCTTTTC_mmu-mir-128-2
47_TTTCAGTCGGATGTTTGC_mmu-mir-30a
47_TTTCAGTCGGATGTTTGCAG_mmu-mir-30a
47_TTTCAGTCGGATGTTTGCAGC_mmu-mir-30a
48_AGCTACATCTGGCTACTGGGTC_mmu-mir-222
48_AGCTACATCTGGCTACTGGGTCT_mmu-mir-222
48_AGCTACATCTGGCTACTGGGTCTC_mmu-mir-222

48_AGCTACATCTGGCTACTGGGTCTCA_mmu-mir-222
48_AGCTACATCTGGCTACTGGGTCTCC_mmu-mir-222
48_AGCTACATCTGGCTACTGGGTCTCT_mmu-mir-222
48_GCTGCACTTGGATTTCGTTC_mmu-mir-191
48_TATACAAGGGCAAGCTCTCTGC_mmu-mir-381
48_TGAAATGTTTAGGACCAC_mmu-mir-203
48_TGAAATGTTTAGGACCACT_mmu-mir-203
48_TGAAATGTTTAGGACCACTAG_mmu-mir-203
48_TGAAATGTTTAGGACCACTAT_mmu-mir-203
48_TGGAATGTAAAGAAGTATGT_mmu-mir-1a-1
48_TTCACAGTGGCTAAGTTCCA_mmu-mir-27b
48_TTCACAGTGGCTAAGTTCCGA_mmu-mir-27b
48_TTCACAGTGGCTAAGTTCCGT_mmu-mir-27b
48_TTCACAGTGGCTAAGTTCT_mmu-mir-27b
48_TTCACAGTGGCTAAGTTCTG_mmu-mir-27b
48_TTCACAGTGGCTAAGTTCTGA_mmu-mir-27b
48_TTCACAGTGGCTAAGTTCTGAAA_mmu-mir-27b
48_TTCACAGTGGCTAAGTTCTGCA_mmu-mir-27b
48_TTCACAGTGGCTAAGTTCTGCAA_mmu-mir-27b
48_TTCACAGTGGCTAAGTTCTGCATT_mmu-mir-27b
48_TTCACAGTGGCTAAGTTCTGCT_mmu-mir-27b
48_TTCACAGTGGCTAAGTTCTGCTC_mmu-mir-27b
48_TTCACAGTGGCTAAGTTCTGCTT_mmu-mir-27b
48_TTCACAGTGGCTAAGTTCTGT_mmu-mir-27b
49_GTACAGTACTGTGATAACT_mmu-mir-101a
49_GTACAGTACTGTGATAACTGA_mmu-mir-101a
49_GTACAGTACTGTGATAACTGAA_mmu-mir-101a
49_TATTGCACTTGTCCCGGCC_mmu-mir-92a-1
49_TATTGCACTTGTCCCGGCCTGC_mmu-mir-92a-1
49_TATTGCACTTGTCCCGGCCTGT_mmu-mir-92a-1
49_TATTGCACTTGTCCCGGCCTGTA_mmu-mir-92a-1
49_TATTGCACTTGTCCCGGCCTGTAA_mmu-mir-92a-1
49_TATTGCACTTGTCCCGGCCTGTT_mmu-mir-92a-1
5_ACCCTGTAGAACCGAATTTGTGA_mmu-mir-10b
5_ACCCTGTAGAACCGAATTTGTGT_mmu-mir-10b
5_CAGTGGTTTTACCCTATGGTA_mmu-mir-140
5_CAGTGGTTTTACCCTATGGTAA_mmu-mir-140
5_CCCAGTGTTCAGACTACCTGTT_mmu-mir-199a-1
5_TAGCAGCGGGAACAGTACTG_mmu-mir-503
5_TATGGCACTGGTAGAATTCACC_mmu-mir-183
5_TATGGCACTGGTAGAATTCACTG_mmu-mir-183
5_TATTGCACATTACTAAGTTGC_mmu-mir-32
5_TCCCTGAGACCCTTTAACCTGC_mmu-mir-125a
5_TCCCTGAGACCCTTTAACCTGT_mmu-mir-125a
5_TCCCTGAGACCCTTTAACCTGTG_mmu-mir-125a
5_TCTCCCAACCCTTGTACC_mmu-mir-150
5_TCTCCCAACCCTTGTACCAG_mmu-mir-150
5_TCTCCCAACCCTTGTACCAGT_mmu-mir-150
5_TCTCCCAACCCTTGTACCAGTA_mmu-mir-150
5_TCTCCCAACCCTTGTACCAGTGC_mmu-mir-150

5_TCTCCCAACCCTTGTACCAGTGT_mmu-mir-150
5_TCTCCCAACCCTTGTACT_mmu-mir-150
5_TCTGCAGCTGTTAAGGATGGTG_mmu-mir-1968
5_TGAGAACTGAATTCCATAGGCTA_mmu-mir-146a
5_TGAGAACTGAATTCCATAGGCTAT_mmu-mir-146a
5_TGAGAACTGAATTCCATG_mmu-mir-146a
5_TGAGAACTGAATTCCATGGG_mmu-mir-146a
5_TGAGAACTGAATTCCATGGGAA_mmu-mir-146a
5_TGAGAACTGAATTCCATGGGT_mmu-mir-146a
5_TGAGAACTGAATTCCATGGGTTA_mmu-mir-146a
5_TGAGAACTGAATTCCATGGGTTT_mmu-mir-146a
5_TGAGAACTGAATTCCATGGGTTTT_mmu-mir-146a
5_TGAGGTAGTAGTTTGTGC_mmu-let-7i
5_TGAGGTAGTAGTTTGTGCC_mmu-let-7i
5_TGAGGTAGTAGTTTGTGCT_mmu-let-7i
5_TGAGGTAGTAGTTTGTGCTGTC_mmu-let-7i
5_TGAGGTAGTAGTTTGTGCTGTTA_mmu-let-7i
5_TGAGGTAGTAGTTTGTGCTGTTT_mmu-let-7i
5_TGGAGTGTGACAATGGTGTTC_mmu-mir-122
5_TGGAGTGTGACAATGGTGTTT_mmu-mir-122
5_TGGAGTGTGACAATGGTGTTTGA_mmu-mir-122
5_TGGAGTGTGACAATGGTGTTTGC_mmu-mir-122
5_TGGAGTGTGACAATGGTGTTTGT_mmu-mir-122
5_TGGTAGACTATGGAACGTAG_mmu-mir-379
5_TGTAAACATCCCCGACTGGAAA_mmu-mir-30a
5_TGTAAACATCCCCGACTGGAAT_mmu-mir-30a
5_TGTAAACATCCTCGACT_mmu-mir-30a
5_TGTAAACATCCTCGACTG_mmu-mir-30a
5_TGTAAACATCCTCGACTGG_mmu-mir-30a
5_TGTAAACATCCTCGACTGGA_mmu-mir-30a
5_TGTAAACATCCTCGACTGGAA_mmu-mir-30a
5_TGTAAACATCCTCGACTGGAAGC_mmu-mir-30a
5_TGTAAACATCCTCGACTGGAAGCT_mmu-mir-30a
5_TGTAAACATCCTCGACTGGAAGCTTT_mmu-mir-30a
5_TTATCAGAATCTCCAGGGG_mmu-mir-361
5_TTCCCTTTGTCATCCTATGCCC_mmu-mir-204
5_TTCCCTTTGTCATCCTATGCCTG_mmu-mir-204
5_TTCCCTTTGTCATCCTATGCCTGT_mmu-mir-204
5_TTCCCTTTGTCATCCTATGCCTT_mmu-mir-204
50_CCGACTTCTGGGCTCCGGCTT_mmu-mir-1964
50_CCGACTTCTGGGCTCCGGCTTC_mmu-mir-1964
50_CCGACTTCTGGGCTCCGGCTTTA_mmu-mir-1964
50_CCGACTTCTGGGCTCCGGCTTTT_mmu-mir-1964
50_CCGACTTCTGGGCTCCGGCTTTTT_mmu-mir-1964
50_TAATACTGTCTGGTAATGCCA_mmu-mir-429
50_TAATACTGTCTGGTAATGCCG_mmu-mir-429
50_TAATACTGTCTGGTAATGCCGC_mmu-mir-429
50_TACAGTACTGTGATAACTGA_mmu-mir-101a
50_TACAGTACTGTGATAACTGAAG_mmu-mir-101a
50_TACAGTACTGTGATAACTGAAGT_mmu-mir-101a

50_TACAGTACTGTGATAACTGACT_mmu-mir-101a
51_AGCAGCATTGTACAGGGCTATG_mmu-mir-103-1
51_CATTGCACTTGTCTCGGTC_mmu-mir-25
51_CATTGCACTTGTCTCGGTCTG_mmu-mir-25
51_CCGCACTGTGGGTACTTGC_mmu-mir-106b
51_CCGCACTGTGGGTACTTGCT_mmu-mir-106b
51_CCGCACTGTGGGTACTTGCTG_mmu-mir-106b
51_CCGCACTGTGGGTACTTGCTGA_mmu-mir-106b
51_CCGCACTGTGGGTACTTGCTGT_mmu-mir-106b
52_AGCGCCTCGGCGACAGAGCCG_mmu-mir-339
52_CTAGCACCATCTGAAATC_mmu-mir-29a
52_CTAGCACCATCTGAAATCGG_mmu-mir-29a
52_CTAGCACCATCTGAAATCGGTC_mmu-mir-29a
52_CTAGCACCATCTGAAATCGGTT_mmu-mir-29a
53_CACTAGATTGTGAGCTGCTGG_mmu-mir-28a
53_CACTAGATTGTGAGCTGCTGGAA_mmu-mir-28a
53_TAACACTGTCTGGTAACGA_mmu-mir-200a
53_TAACACTGTCTGGTAACGATA_mmu-mir-200a
53_TAACACTGTCTGGTAACGATG_mmu-mir-200a
53_TAACACTGTCTGGTAACGATGTT_mmu-mir-200a
53_TAGCACCATCTGAAAT_mmu-mir-29a
53_TAGCACCATCTGAAATC_mmu-mir-29a
53_TAGCACCATCTGAAATCGA_mmu-mir-29a
53_TAGCACCATCTGAAATCGG_mmu-mir-29a
53_TAGCACCATCTGAAATCGGT_mmu-mir-29a
53_TAGCACCATCTGAAATCGGTT_mmu-mir-29a
54_ACGGGTTAGGCTCTTGGGAGC_mmu-mir-125b-1
54_CCGTCCTGAGGTTGTTGAGCTG_mmu-mir-676
54_CCGTCCTGAGGTTGTTGAGCTT_mmu-mir-676
54_CCGTCCTGAGGTTGTTGAGCTTT_mmu-mir-676
54_TATTGCACTTGTCCCGGCCTGA_mmu-mir-92a-2
54_TATTGCACTTGTCCCGGCCTGAA_mmu-mir-92a-2
54_TATTGCACTTGTCCCGGCCTGATT_mmu-mir-92a-2
54_TATTGCACTTGTCCCGGCCTGC_mmu-mir-92a-2
54_TATTGCACTTGTCCCGGCCTGTA_mmu-mir-92a-2
55_TTCACAGTGGCTAAGTTC_mmu-mir-27a
55_TTCACAGTGGCTAAGTTCA_mmu-mir-27a
55_TTCACAGTGGCTAAGTTCC_mmu-mir-27a
55_TTCACAGTGGCTAAGTTCCA_mmu-mir-27a
55_TTCACAGTGGCTAAGTTCCG_mmu-mir-27a
55_TTCACAGTGGCTAAGTTCCGA_mmu-mir-27a
55_TTCACAGTGGCTAAGTTCCGAT_mmu-mir-27a
55_TTCACAGTGGCTAAGTTCCGCT_mmu-mir-27a
55_TTCACAGTGGCTAAGTTCCGT_mmu-mir-27a
55_TTCACAGTGGCTAAGTTCCGTT_mmu-mir-27a
55_TTTTTCATTATTGCTCCTGACT_mmu-mir-335
56_AAGCTGCCAGTTGAAGAAC_mmu-mir-22
56_AAGCTGCCAGTTGAAGAACC_mmu-mir-22
56_AAGCTGCCAGTTGAAGAACG_mmu-mir-22
56_AAGCTGCCAGTTGAAGAACT_mmu-mir-22

56_AAGCTGCCAGTTGAAGAACTGA_mmu-mir-22
56_AAGCTGCCAGTTGAAGAACTGC_mmu-mir-22
58_CCAATATTGGCTGTGCTGCTC_mmu-mir-195a
58_CCAATATTGGCTGTGCTGCTCCA_mmu-mir-195a
58_CCAATATTGGCTGTGCTGCTCT_mmu-mir-195a
58_CTTTCAGTCGGATGTTTAC_mmu-mir-30e
58_CTTTCAGTCGGATGTTTACAG_mmu-mir-30e
58_TTTGGTCCCCTTCAACCAGCTGT_mmu-mir-133a-2
59_AGCTACATTGTCTGCTGGGTT_mmu-mir-221
59_AGCTACATTGTCTGCTGGGTTT_mmu-mir-221
59_AGCTACATTGTCTGCTGGGTTTCT_mmu-mir-221
59_CACAGCTCCCATCTCAGAAA_mmu-mir-674
59_CACAGCTCCCATCTCAGAAC_mmu-mir-674
59_CACAGCTCCCATCTCAGAACA_mmu-mir-674
59_CACAGCTCCCATCTCAGAACAAT_mmu-mir-674
59_CACAGCTCCCATCTCAGAAT_mmu-mir-674
59_CCTCTGGGCCCTTCCTCCAG_mmu-mir-326
59_GTACAGTACTGTGATAGCTG_mmu-mir-101b
59_GTACAGTACTGTGATAGCTGA_mmu-mir-101b
59_TCCGAGCCTGGGTCTCCC_mmu-mir-615
59_TCCGAGCCTGGGTCTCCCT_mmu-mir-615
59_TCCGAGCCTGGGTCTCCCTC_mmu-mir-615
59_TCCGAGCCTGGGTCTCCCTCT_mmu-mir-615
59_TTGGTCCCCTTCAACCAGCTGT_mmu-mir-133a-2
59_TTTGAACCATCACTCGACTCC_mmu-mir-434
6_AACATTCAACGCTGTCGGT_mmu-mir-181a-2
6_AACATTCAACGCTGTCGGTA_mmu-mir-181a-2
6_AACATTCAACGCTGTCGGTG_mmu-mir-181a-2
6_AACATTCAACGCTGTCGGTGA_mmu-mir-181a-2
6_AACATTCATTGTTGTCGGTG_mmu-mir-181d
6_AACATTCATTGTTGTCGGTGG_mmu-mir-181d
6_AACATTCATTGTTGTCGGTGGG_mmu-mir-181d
6_ATGGCACTGGTAGAATTCACT_mmu-mir-183
6_ATGGCACTGGTAGAATTCACTG_mmu-mir-183
6_CAAAGAATTCTCCTTTTGGA_mmu-mir-186
6_CAAAGAATTCTCCTTTTGGG_mmu-mir-186
6_CAAAGAATTCTCCTTTTGGGC_mmu-mir-186
6_CAAAGAATTCTCCTTTTGGGCA_mmu-mir-186
6_CAAAGAATTCTCCTTTTGGGCC_mmu-mir-186
6_CAAAGAATTCTCCTTTTGGGT_mmu-mir-186
6_CAACGGAATCCCAAAAGC_mmu-mir-191
6_CAACGGAATCCCAAAAGCAGCC_mmu-mir-191
6_CAACGGAATCCCAAAAGCAGCT_mmu-mir-191
6_CAACGGAATCCCAAAAGCAGCTA_mmu-mir-191
6_CAACGGAATCCCAAAAGT_mmu-mir-191
6_CACCCGTAGAACCGACCTTG_mmu-mir-99b
6_CACCCGTAGAACCGACCTTGA_mmu-mir-99b
6_CACCCGTAGAACCGACCTTGC_mmu-mir-99b
6_CACCCGTAGAACCGACCTTGCGA_mmu-mir-99b
6_CACCCGTAGAACCGACCTTGCGT_mmu-mir-99b

6_CACCCGTAGAACCGACCTTGT_mmu-mir-99b
6_TCCCTGAGACCCTAACTTG_mmu-mir-125b-2
6_TCCCTGAGACCCTAACTTGC_mmu-mir-125b-2
6_TCCCTGAGACCCTAACTTGT_mmu-mir-125b-2
6_TCCCTGAGACCCTAACTTGTG_mmu-mir-125b-2
6_TCCCTGAGACCCTAACTTGTGT_mmu-mir-125b-2
6_TCCTTCATTCCACCGGAGTCTGA_mmu-mir-205
6_TCCTTCATTCCACCGGAGTCTGT_mmu-mir-205
6_TCTACAGTGCACGTGTCTCC_mmu-mir-139
6_TCTACAGTGCACGTGTCTCCA_mmu-mir-139
6_TCTACAGTGCACGTGTCTCCAA_mmu-mir-139
6_TCTACAGTGCACGTGTCTCCAGA_mmu-mir-139
6_TCTACAGTGCACGTGTCTCCAGT_mmu-mir-139
6_TCTACAGTGCACGTGTCTCCAGTT_mmu-mir-139
6_TCTACAGTGCACGTGTCTCCAT_mmu-mir-139
6_TCTACAGTGCACGTGTCTCT_mmu-mir-139
6_TGAGGTAGTAGGTTGTATGGTC_mmu-let-7b
6_TGAGGTAGTAGGTTGTATGGTTA_mmu-let-7b
6_TGAGGTAGTAGGTTGTG_mmu-let-7b
6_TGAGGTAGTAGGTTGTGT_mmu-let-7b
6_TGAGGTAGTAGGTTGTGTA_mmu-let-7b
6_TGAGGTAGTAGGTTGTGTG_mmu-let-7b
6_TGAGGTAGTAGGTTGTGTGA_mmu-let-7b
6_TGAGGTAGTAGGTTGTGTGG_mmu-let-7b
6_TGAGGTAGTAGGTTGTGTGGC_mmu-let-7b
6_TGAGGTAGTAGGTTGTGTGGT_mmu-let-7b
6_TGAGGTAGTAGGTTGTGTGGTAA_mmu-let-7b
6_TGAGGTAGTAGGTTGTGTGGTAT_mmu-let-7b
6_TGAGGTAGTAGGTTGTGTGGTC_mmu-let-7b
6_TGAGGTAGTAGGTTGTGTGGTTA_mmu-let-7b
6_TGAGGTAGTAGGTTGTGTGGTTAA_mmu-let-7b
6_TGAGGTAGTAGGTTGTGTGGTTAC_mmu-let-7b
6_TGAGGTAGTAGGTTGTGTGGTTAT_mmu-let-7b
6_TGAGGTAGTAGGTTGTGTGGTTT_mmu-let-7b
6_TGAGGTAGTAGGTTGTGTGGTTTT_mmu-let-7b
6_TGAGGTAGTAGGTTGTGTGGTTTTT_mmu-let-7b
6_TGAGGTAGTAGGTTGTGTGT_mmu-let-7b
6_TGAGGTAGTAGTTTGTAC_mmu-let-7g
6_TGAGGTAGTAGTTTGTACAGC_mmu-let-7g
6_TGAGGTAGTAGTTTGTACAGT_mmu-let-7g
6_TGAGGTAGTAGTTTGTACAGTC_mmu-let-7g
6_TGAGGTAGTAGTTTGTACAGTTA_mmu-let-7g
6_TGAGGTAGTAGTTTGTACAGTTT_mmu-let-7g
6_TGAGGTAGTAGTTTGTGCCGTT_mmu-let-7g
6_TGAGGTAGTAGTTTGTGCTGTTT_mmu-let-7g
6_TGAGGTAGTAGTTTGTGTTGTT_mmu-let-7b
6_TGGAGAGAAAGGCAGTTCCTG_mmu-mir-185
6_TGTAACAGCAACTCCATGTGG_mmu-mir-194-1
6_TTTGGCAATGGTAGAACTCACA_mmu-mir-182
6_TTTGGCAATGGTAGAACTCACACA_mmu-mir-182

6_TTTGGCAATGGTAGAACTCACACC_mmu-mir-182
6_TTTGGCAATGGTAGAACTCACACT_mmu-mir-182
60_AAACATGAAGCGCTGCAACA_mmu-mir-322
60_AATGCACCCGGGCAAGGATT_mmu-mir-501
60_AATGCACCCGGGCAAGGATTTGG_mmu-mir-501
60_CTATACAACCTACTGCCTTCCT_mmu-let-7b
60_CTGGCCCTCTCTGCCCTT_mmu-mir-328
60_CTGGCCCTCTCTGCCCTTC_mmu-mir-328
60_CTGGCCCTCTCTGCCCTTCCA_mmu-mir-328
60_CTGGCCCTCTCTGCCCTTCCG_mmu-mir-328
60_CTGGCCCTCTCTGCCCTTCCGA_mmu-mir-328
60_CTGGCCCTCTCTGCCCTTCCGC_mmu-mir-328
60_CTGGCCCTCTCTGCCCTTCCGTT_mmu-mir-328
60_GCAAAGCACAGGGCCTGCAGAGAGT_mmu-mir-330
60_TACAGTACTGTGATAGCTGA_mmu-mir-101b
60_TACAGTACTGTGATAGCTGAAG_mmu-mir-101b
60_TACAGTACTGTGATAGCTGAAGT_mmu-mir-101b
60_TCAGTGCACAACAGAACTTTGT_mmu-mir-148b
60_TCAGTGCACCACAGAACTTTGT_mmu-mir-148b
60_TCAGTGCACTACAGAACT_mmu-mir-148a
60_TCAGTGCACTACAGAACTT_mmu-mir-148a
60_TCAGTGCACTACAGAACTTGT_mmu-mir-148a
60_TCAGTGCACTACAGAACTTT_mmu-mir-148a
60_TCAGTGCACTACAGAACTTTGC_mmu-mir-148a
60_TCAGTGCATCACAGAACTTTGC_mmu-mir-148b
60_TCAGTGCATCACAGAACTTTGTT_mmu-mir-148b
60_TCCGTCTCAGTTACTTTATAG_mmu-mir-340
60_TCTCACACAGAAATCGCACCCGTC_mmu-mir-342
60_TGGCTCAGTTCAGCAGGAAC_mmu-mir-24-2
60_TGGCTCAGTTCAGCAGGAACA_mmu-mir-24-2
61_CAAAGCACAGGGCCTGCAGAGAGT_mmu-mir-330
61_CTATACAATCTACTGTCTTTCT_mmu-let-7c-2
62_AGTGCACTACAGAACTTTGT_mmu-mir-148a
63_AAGCTCGGTCTGAGGCCCCTC_mmu-mir-423
63_AAGCTCGGTCTGAGGCCCCTCA_mmu-mir-423
63_CTATACAATCTACTGTCTTTCT_mmu-let-7a-1
63_TACAGTAGTCTGCACATTGGTT_mmu-mir-199b
64_ACAGTAGTCTGCACATTGGT_mmu-mir-199b
64_ACAGTAGTCTGCACATTGGTT_mmu-mir-199b
64_AGCTCGGTCTGAGGCCCCT_mmu-mir-423
64_AGCTCGGTCTGAGGCCCCTC_mmu-mir-423
64_AGCTCGGTCTGAGGCCCCTCA_mmu-mir-423
64_AGCTCGGTCTGAGGCCCCTCAG_mmu-mir-423
64_AGCTCGGTCTGAGGCCCCTCAGA_mmu-mir-423
64_AGCTCGGTCTGAGGCCCCTCAGC_mmu-mir-423
64_AGCTCGGTCTGAGGCCCCTCAGTT_mmu-mir-423
64_AGCTCGGTCTGAGGCCCCTT_mmu-mir-423
65_CAGTAGTCTGCACATTGGT_mmu-mir-199b
65_CAGTAGTCTGCACATTGGTT_mmu-mir-199b
65_CTGTGCGTGTGACAGCGGCTGAAA_mmu-mir-210

67_TGTCAGTTTGTCAAATACCCCAT_mmu-mir-223
67_TGTCAGTTTGTCAAATACCCCATT_mmu-mir-223
67_TGTCAGTTTGTCAAATACCCCT_mmu-mir-223
68_TACAGTAGTCTGCACATTGGTT_mmu-mir-199a-2
69_ACAGTAGTCTGCACATTGGT_mmu-mir-199a-2
69_ACAGTAGTCTGCACATTGGTC_mmu-mir-199a-2
69_ACAGTAGTCTGCACATTGGTT_mmu-mir-199a-2
69_CTATACGACCTGCTGCCTTT_mmu-let-7d
69_CTATACGACCTGCTGCCTTTC_mmu-let-7d
69_CTATACGACCTGCTGCCTTTCA_mmu-let-7d
69_TACAGCAGGCACAGACAGA_mmu-mir-214
69_TACAGCAGGCACAGACAGG_mmu-mir-214
69_TACAGCAGGCACAGACAGGA_mmu-mir-214
69_TACAGCAGGCACAGACAGGAAT_mmu-mir-214
7_TAAGTCACTAGTGGTTCCGTTT_mmu-mir-224
7_TAAGTCACTAGTGGTTCCGTTTAG_mmu-mir-224
7_TCGAGGAGCTCACAGTCT_mmu-mir-151
7_TCGAGGAGCTCACAGTCTAGC_mmu-mir-151
7_TCGAGGAGCTCACAGTCTAGTA_mmu-mir-151
7_TGAGGTAGTAGATT_mmu-let-7f-2
7_TGAGGTAGTAGATTGTAT_mmu-let-7f-1
7_TGAGGTAGTAGATTGTAT_mmu-let-7f-2
7_TGAGGTAGTAGATTGTATA_mmu-let-7f-2
7_TGAGGTAGTAGATTGTATAG_mmu-let-7f-1
7_TGAGGTAGTAGATTGTATAG_mmu-let-7f-2
7_TGAGGTAGTAGATTGTATAGC_mmu-let-7f-2
7_TGAGGTAGTAGATTGTATAGT_mmu-let-7f-1
7_TGAGGTAGTAGATTGTATAGT_mmu-let-7f-2
7_TGAGGTAGTAGATTGTATAGTC_mmu-let-7f-1
7_TGAGGTAGTAGATTGTATAGTC_mmu-let-7f-2
7_TGAGGTAGTAGATTGTATAGTTC_mmu-let-7f-1
7_TGAGGTAGTAGATTGTATAGTTC_mmu-let-7f-2
7_TGAGGTAGTAGATTGTATAGTTT_mmu-let-7f-2
7_TGAGGTAGTAGGCTGTATAGTT_mmu-let-7f-2
7_TGAGGTAGTAGGTTGTATAGC_mmu-let-7f-2
7_TGAGGTAGTAGGTTGTATAGTC_mmu-let-7f-1
7_TGAGGTAGTAGGTTGTATAGTC_mmu-let-7f-2
7_TGCAGCTGTTAAGGATGGTGGA_mmu-mir-1968
70_ACAGCAGGCACAGACAGGAAA_mmu-mir-214
70_CAGTAGTCTGCACATTGGTT_mmu-mir-199a-2
72_CAACTAGACTGTGAGCTTCT_mmu-mir-708
72_CAACTAGACTGTGAGCTTCTA_mmu-mir-708
74_CGGGGCAGCTCAGTACAGGATG_mmu-mir-486a
8_ATCCCACTTCTGACACCA_mmu-mir-3963
8_CATCTTACTGGGCAGCATTGG_mmu-mir-200b
9_TGAGGTTGTAGTTTGTGCTGTT_mmu-mir-3970
